# The H2A.Z and NuRD associated protein HMG20A controls early head and heart developmental transcription programs

**DOI:** 10.1038/s41467-023-36114-x

**Published:** 2023-01-28

**Authors:** Andreas Herchenröther, Stefanie Gossen, Tobias Friedrich, Alexander Reim, Nadine Daus, Felix Diegmüller, Jörg Leers, Hakimeh Moghaddas Sani, Sarah Gerstner, Leah Schwarz, Inga Stellmacher, Laura Victoria Szymkowiak, Andrea Nist, Thorsten Stiewe, Tilman Borggrefe, Matthias Mann, Joel P. Mackay, Marek Bartkuhn, Annette Borchers, Jie Lan, Sandra B. Hake

**Affiliations:** 1 Institute for Genetics, Justus-Liebig University Giessen, Giessen, Germany; 2 Department of Biology, Molecular Embryology, Philipps University Marburg, Marburg, Germany; 3 Institute for Biochemistry, Justus-Liebig University Giessen, Giessen, Germany; 4 Biomedical Informatics and Systems Medicine, Science Unit for Basic and Clinical Medicine, Institute for lung health, Justus-Liebig University Giessen, Giessen, Germany; 5 Department of Proteomics and Signal Transduction, Max-Planck Institute of Biochemistry, Martinsried, Germany; 6 School of Life and Environmental Sciences, University of Sydney, New South Wales, Australia; 7 Institute for Physiological Chemistry, Technical University Dresden, Dresden, Germany; 8 Genomics Core Facility, Institute of Molecular Oncology, Universities of Giessen and Marburg Lung Center, Member of the German Center for Lung Research (DZL), Philipps-University Marburg, Marburg, Germany

**Keywords:** Histone variants, Differentiation, Organogenesis

## Abstract

Specialized chromatin-binding proteins are required for DNA-based processes during development. We recently established PWWP2A as a direct histone variant H2A.Z interactor involved in mitosis and craniofacial development. Here, we identify the H2A.Z/PWWP2A-associated protein HMG20A as part of several chromatin-modifying complexes, including NuRD, and show that it localizes to distinct genomic regulatory regions. Hmg20a depletion causes severe head and heart developmental defects in *Xenopus laevis*. Our data indicate that craniofacial malformations are caused by defects in neural crest cell (NCC) migration and cartilage formation. These developmental failures are phenocopied in *Hmg20a*-depleted mESCs, which show inefficient differentiation into NCCs and cardiomyocytes (CM). Consequently, loss of HMG20A, which marks open promoters and enhancers, results in chromatin accessibility changes and a striking deregulation of transcription programs involved in epithelial-mesenchymal transition (EMT) and differentiation processes. Collectively, our findings implicate HMG20A as part of the H2A.Z/PWWP2A/NuRD-axis and reveal it as a key modulator of intricate developmental transcription programs that guide the differentiation of NCCs and CMs.

## Abstract (web summary)

How the histone variant H2A.Z controls cell fate remains unclear. Here, the authors reveal that the H2A.Z interacting partner HMG20A plays a key role in regulating transcription during early head and heart development.

## Introduction

Proper control of chromatin structure is important for the regulation of eukaryotic gene expression, which is necessary for successful embryonic development and efficient stem cell differentiation. This complex process includes the incorporation of histone variants into chromatin, ATP-dependent remodelling of nucleosomes, the action of regulatory RNAs, and chemical modifications of DNA and histone proteins. How histone variants, as important factors controlling the accessibility of the underlying genetic information^1^, mechanistically coordinate gene regulation is still not fully understood.

The histone variant H2A.Z is highly conserved^2^ and in vertebrates encoded by two genes (*H2AFZ* and *H2AFV*), whose protein products (H2A.Z.1 and H2A.Z.2.1) differ in only three amino acids^3^. In primates, the *H2AFV* RNA can be alternatively spliced, giving rise to an additional H2A.Z.2.2 protein with a shortened, nucleosome-destabilizing C-terminus^4,5^. Importantly, H2A.Z has been shown to be essential for embryogenesis and is involved in neurodevelopmental processes^2,6–8^. However, the underlying molecular mechanism(s) remains ill-defined, although H2A.Z has been implicated in many DNA-based processes including transcriptional regulation, cell cycle control, and DNA repair^8–13^.

Together with recent studies, our previous work has shed some light on the functional network of H2A.Z in gene regulation and embryonic development^14^, where the interactomes of H2A.Z isoforms have been determined. We discovered, among several other proteins, PWWP2A as a direct and highly specific binder of H2A.Z.1 and H2A.Z.2.1 nucleosomes^15,16^. Depletion of PWWP2A causes severe phenotypes, such as significant delays in mitotic progression in human cell lines and strong craniofacial defects in *Xenopus laevis*, most likely due to defects in neural crest cell (NCC) migration and differentiation^15,17^. We found that PWWP2A regulates many H2A.Z-controlled genes, probably by recruiting chromatin-modifying proteins, such as an MTA1-specific core nucleosome and remodelling and deacetylase (NuRD) complex (M1HR). M1HR lacks the remodelling CHD subunit usually found in NuRD, due to competition between PWWP2A and the MBD proteins that recruit CHD to the NuRD complex^16,18–20^.

In addition to M1HR, PHD Finger protein 14 (PHF14), Retinoic Acid Induced 1 (RAI1), Transcription Factor 20 (TCF20) and High Mobility Group 20 A (HMG20A) proteins were repeatedly identified in both H2A.Z.1/2.1 and PWWP2A interactomes^15,16,21^. A complex, which we termed PRTH because of the initials of the four complex members. These four proteins have previously been shown to be repelled by histone H3 lysine 4 trimethylation (H3K4me3) and to be part of one unified complex^22^. More importantly, all four proteins have been shown to be deregulated or mutated in neurodevelopmental diseases, including intellectual disabilities and autism spectrum disorders^23^, by disrupting the H3K4 methylation signature that ensures normal brain development^24^. As PWWP2A depletion in *Xenopus laevis* results in severe defects in craniofacial development, we wondered whether its association with PHF14/RAI1/TCF20/HMG20A might be – at least partially – the cause of this observation. Hence, we turned our attention to the functional characterization of one member of this complex. In this study, we focus on HMG20A (previously termed iBRAF), as little is known about its histone variant-related function(s), particularly in embryonic development.

Here, we expand the published HMG20A interactome, taking advantage of GFP-HMG20A expressing human cells, and identify, besides BHC/CoREST and PRTH proteins, members of the NuRD complex. Subsequently, we demonstrate that binding to these proteins depends on the C-terminal region of HMG20A, which contains a coiled-coil (CC) domain, while the N-terminus with the conserved HMG box conveys DNA-binding activity. Furthermore, ChIP-seq experiments reveal a strong enrichment of HMG20A in two separate genomic regions: nucleosome-depleted transcriptional start sites (TSSs) surrounded by H2A.Z/PWWP2A-containing nucleosomes, and H2A.Z/PWWP2A-lacking intronic enhancer regions. Due to a high conservation of HMG20A between humans, mice, and *Xenopus*, we next determined the biological significance of HMG20A using *Xenopus laevis* wherein depletion of Hmg20a in vivo resulted in severe defects in craniofacial and heart development. In addition, we observed that both NCC migration and cartilage differentiation processes were impaired, phenotypes that were also observed after loss of Pwwp2a. Similar defects were recapitulated in *Hmg20a*-depleted mouse embryonic stem cells (mESCs), which showed compromised differentiation to NCCs and beating cardiomyocytes (CM). HMG20A loss resulted in chromatin accessibility changes and a striking deregulation of specific transcription programs in NCCs and CMs, including, among others, genes responsible for epithelial-mesenchymal transition (EMT). At the molecular level, we profiled endogenous HMG20A binding sites genome-wide in primed mESCs and revealed the enrichment of HMG20A at H2A.Z-surrounded promoter as well as regulatory regions devoid of H2A.Z, similar to our findings in human cells. Furthermore, genes associated only with HMG20A are enriched with GO terms such as ‘embryo morphogenesis’, while genes associated with HMG20A and H2A.Z belong to ‘chromatin organization’ functions.

In summary, our study identifies HMG20A as a chromatin binding protein that acts in conjunction with several chromatin-modifying complexes and that resides in promoter and enhancer regions. HMG20A serves as a key modulator participating in the regulation of transcription programs important for stem cell differentiation during early development.

## Results

### HMG20A interacts with PRTH, BHC/CoREST, and NuRD complexes

**Fig. 1:**
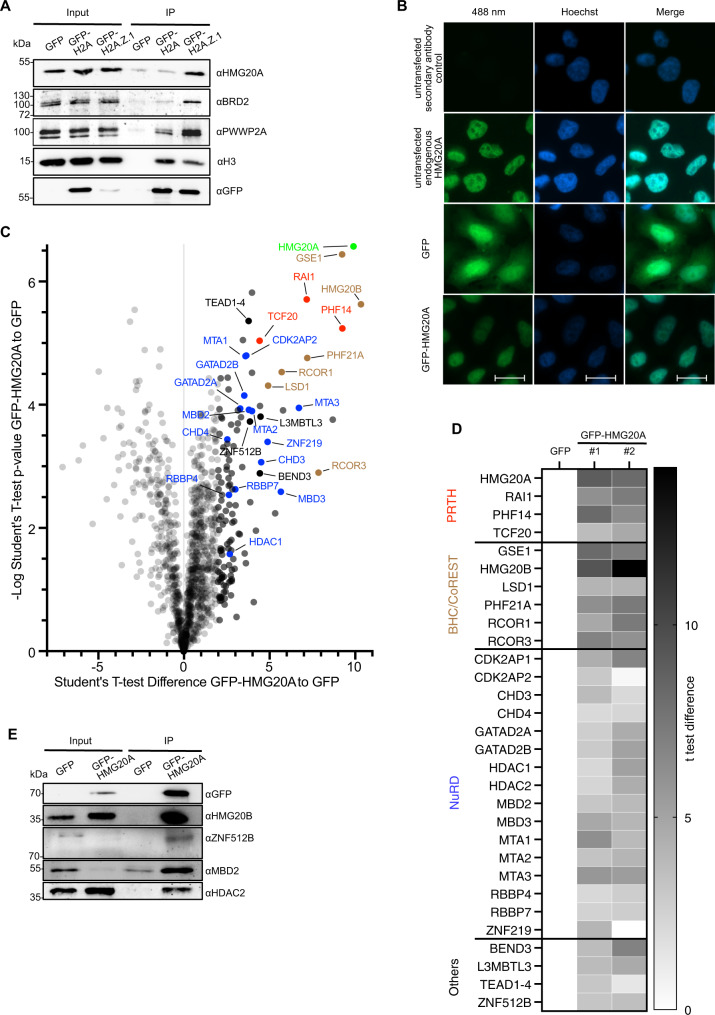
HMG20A binds several chromatin-modifying complexes. **A** Immunoblots of GFP, HMG20A, BRD2 and PWWP2A (positive controls) as well as H3 upon GFP, GFP-H2A and GFP-H2A.Z.1 mononucleosome IPs. **B** Immunofluorescence microscopy images of GFP, GFP-HMG20A and endogenous HMG20A proteins (488 nm, green) in HeLaK cells. DNA is stained with Hoechst (blue). Scale bar: 20 µm. **C** Volcano plot of label-free interaction partners of GFP-HMG20A-associated mononucleosomes. Significantly enriched proteins over GFP-associated mononucleosomes are shown in the upper right part. t-Test (two-tailed) differences were obtained by two-sample t-test. HMG20A is highlighted in bright green, PRTH members in red, BHC/CoREST members in brown, NuRD members in blue, other proteins in black and background binding proteins in grey. See also Supplementary Fig. 1E for Volcano plot of second biological replicate and Supplementary Data 1 for detailed list of HMG20A binders. **D** Heatmap of significant outliers from two independent GFP-HMG20A mononucleosome IPs analysed by lf-qMS/MS (see **C** and Supplementary Fig. 1E) normalized to GFP. Scale bar: log2-fold t-test differences (two-tailed). **E** Immunoblots of GFP and GFP-HMG20A mononucleosome-IPs detecting endogenous members of the BHC/CoREST (HMG20B) and NuRD (MBD2, HDAC2) complexes as well as ZNF512B protein. Experiments in **A**, **B**, **E** were repeated independently three times with consistency. Source data for these figures are provided as a Source Data file.

Previously, we used label-free quantitative mass spectrometry (lf-qMS) approaches to identify H2A.Z- and PWWP2A-mononucleosome binding proteins in HeLa Kyoto (HeLaK) cell lines^15,17,21^. Among several other proteins, we detected the PRTH complex members PHF14, RAI1, TCF20 and HMG20A as strong binders in both H2A.Z and PWWP2A interactome data sets. To gain further insight into any functional H2A.Z connection between these proteins, we focused our attention on HMG20A, which is vertebrate-specific and contains an internal HMG box and a C-terminal coiled-coil (CC) region (Supplementary Fig. 1A). First, we confirmed the association of H2A.Z with HMG20A by performing mononucleosome immunoprecipitations (mononuc-IPs) with HeLaK cells stably expressing GFP-H2A or GFP-H2A.Z.1 (Fig. 1A). Next, we generated HeLaK cell lines stably expressing N-terminally GFP-tagged HMG20A (Supplementary Fig. 1B, C). As expected, GFP-HMG20A was predominantly observed in the nucleus, with a localization pattern similar to that of endogenous HMG20A protein (Fig. 1B). Interestingly, GFP-HMG20A expression somehow led to a reduction of endogenous HMG20A protein, resulting in a total GFP-HMG20A protein level comparable to endogenous HMG20A in non-transfected WT cells (Supplementary Fig. 1C).

To identify the HMG20A interactome, we digested chromatin to mononucleosomes (Supplementary Fig. 1D) and immunoprecipitated GFP- or GFP-HMG20A with GFP-TRAP beads. Bound proteins were subjected to on-bead tryptic digestion and then quantified by lf-qMS/MS. Identification of PRTH members PHF14, RAI1, TCF20 and the BHC/CoREST proteins HMG20B, GSE1, PHF21A, KDM1A, RCOR1 and RCOR3 as documented binders of HMG20A^24,25^ provided confidence in our approach (Fig. 1C, D, Supplementary Fig. 1E, Supplementary Data 1). The interaction of HMG20A with some BHC/CoREST and NuRD members was independently verified by immunoblotting (Fig. 1E). Interestingly, we detected several proteins and subunits of complexes previously identified to interact with H2A.Z and PWWP2A but not uniquely with H2A.Z nucleosomes (Supplementary Fig. 1F), implying that HMG20A could rather be a PWWP2A-associated protein than a direct binder of H2A.Z. Of particular interest was the observation that the complete NuRD complex was reproducibly pulled-down by GFP-HMG20A, as were members of the TEAD transcription factor family, the zinc finger protein ZNF512B and the chromatin modifying factors BEND3 and L3MBTL3 (Fig. 1C–E, Supplementary Fig. 1E, F).

In summary, we identified PRTH, BHC/CoREST, and NuRD complexes, as well as several chromatin-modifying proteins as HMG20A-associated factors. Some of these proteins are also part of the H2A.Z/PWWP2A interactomes (PRTH, M1HR, ZNF512B), while others appear to be HMG20A-specific (BHC/CoREST, NuRD), providing at least two alternative mechanisms for HMG20A to exert its biological function.

### HMG20A’s coiled-coil domain containing C-terminus is sufficient for NuRD binding

**Fig. 2:**
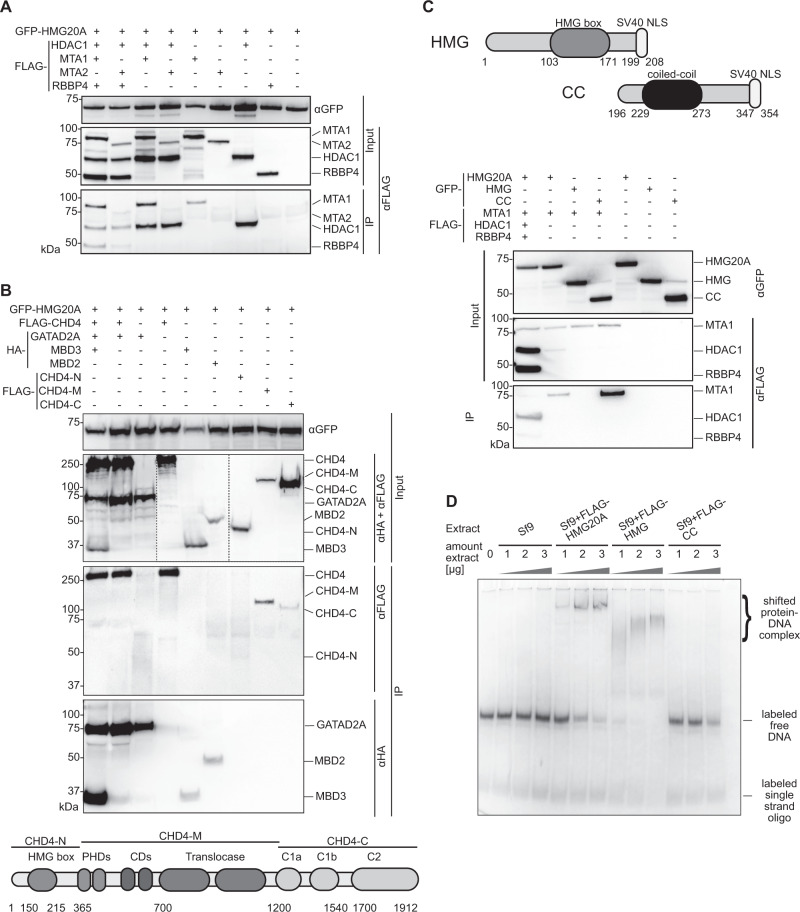
HMG20A binds NuRD complex components and DNA. **A** Anti-GFP immunoprecipitations of HEK293 cell extracts co-transfected with GFP-HMG20A and FLAG-HDAC1, -MTA1, -MTA2 and -RBBP4. Proteins were detected with anti-FLAG or anti-GFP antibodies. **B** Top: Anti-GFP immunoprecipitations of HEK293 cell extracts co-transfected with GFP-HMG20A and FLAG-CHD4 (CHD4), -CHD4-N-terminus (CHD4-N), -CHD4-middle domain (CHD4-M), -CHD4-C-terminus (CHD4-C) and HA-GATAD2A, -MBD2 and -MBD3. Proteins were detected with anti-FLAG and anti-HA or anti-GFP antibodies. Bottom: schematic depiction of CHD4 deletion constructs. **C** Top: schematic depiction of HMG20A deletion constructs. Bottom: anti-GFP IPs of HEK293 cell extracts co-transfected with GFP-HMG20A and its deletions (HMG, CC) and of NuRD members (FLAG-MTA1, HDAC1-FLAG, FLAG-RBBP4). Proteins were detected with anti-FLAG or anti-GFP antibodies. **D** EMSA of increasing amounts of extracts from Sf9 cells expressing FLAG-HMG20A and its deletions (see C, top) using Cy5-labelled DNA. Experiments in A-E were repeated independently at least two times with consistency. Source data for these figures are provided as a Source Data file.

Having discovered that HMG20A interacts with the entire NuRD complex, we next asked how HMG20A contacts this complex. To answer this question, we co-expressed GFP-HMG20A with combinations of FLAG-tagged MTA1, MTA2, RBBP4 and/or HDAC1 in HEK293 cells. Immunoprecipitation of GFP-HMG20A from cell extracts revealed a stronger association with MTA1 than with MTA2 (Fig. 2A). Additionally, we observed strong binding to HDAC1, whereas HMG20A did not interact with RBBP4 alone. We detected interactions of GFP-HMG20A with HA-MBD2/3, HA-GATAD2A and FLAG-CHD4 (Fig. 2B, top), indicating that HMG20A, in contrast to PWWP2A^16,18,20^, pulls down both remodelling and deacetylase subunits of NuRD. Further mapping of the interaction with CHD4 using deletion constructs (Fig. 2B, bottom) revealed that the interaction with HMG20A is mediated by the central DNA translocase domain, rather than the N and C-terminal domains that are known to mediate interactions with nucleosomes, poly-ADP-ribose and GATAD2A/B^26,27^ (Fig. 2B, top). We note that the nature of these pulldown experiments, which use proteins overexpressed in mammalian cells and detected by western blot, makes it difficult to conclude that any given interaction is direct, rather than being mediated by an additional cellular protein. However, the negative results observed for NuRD subunits such as RBBP4 provide confidence that we are not simply observing indirect interactions mediated by endogenous NuRD subunits.

Next, we asked which region of HMG20A is required for NuRD binding. Like previously reported for the BHC/CoREST complex^28^, it is HMG20A’s C-terminal region containing the CC domain but not the HMG box-containing N-terminus (Fig. 2C, top) that is required for its binding to MTA1 when expressed together with combinations of FLAG-tagged MTA1, RBBP4, and HDAC1 in HEK293 cells (Fig. 2C, bottom).

Lastly, we wondered whether the HMG box from HMG20A retains its ability to bind DNA, since it harbours the conserved amino acids known to be required for the recognition of nucleic acids by HMG domains^29^ (Supplementary Fig. 2A). To test this, we expressed FLAG-HMG20A and the corresponding deletion constructs (Fig. 2C, top) in Sf9 cells (Supplementary Fig. 2B, C) and performed Electromobility Shift Assays (EMSAs) using a Cy5-labelled, 100-bp random DNA probe. As expected, the N-terminal domain of HMG20A that contains the HMG box is indeed capable of binding free DNA (Fig. 2D), in agreement with recent reports in which the full-length HMG20A protein was used^30^ or in which the HMG box was demonstrated to bind with high affinity to double-stranded, four-way-junction DNA^31^.

Together, these results reveal details of the interaction between HMG20A and NuRD components (CHD4 and MTA1) and further highlight the conserved functions of HMG20A’s N-terminal and C-terminal regions in DNA binding and protein-protein interaction, respectively.

### HMG20A localizes to H2A.Z/PWWP2A-containing regulatory regions and intronic enhancer regions lacking H2A.Z

**Fig. 3:**
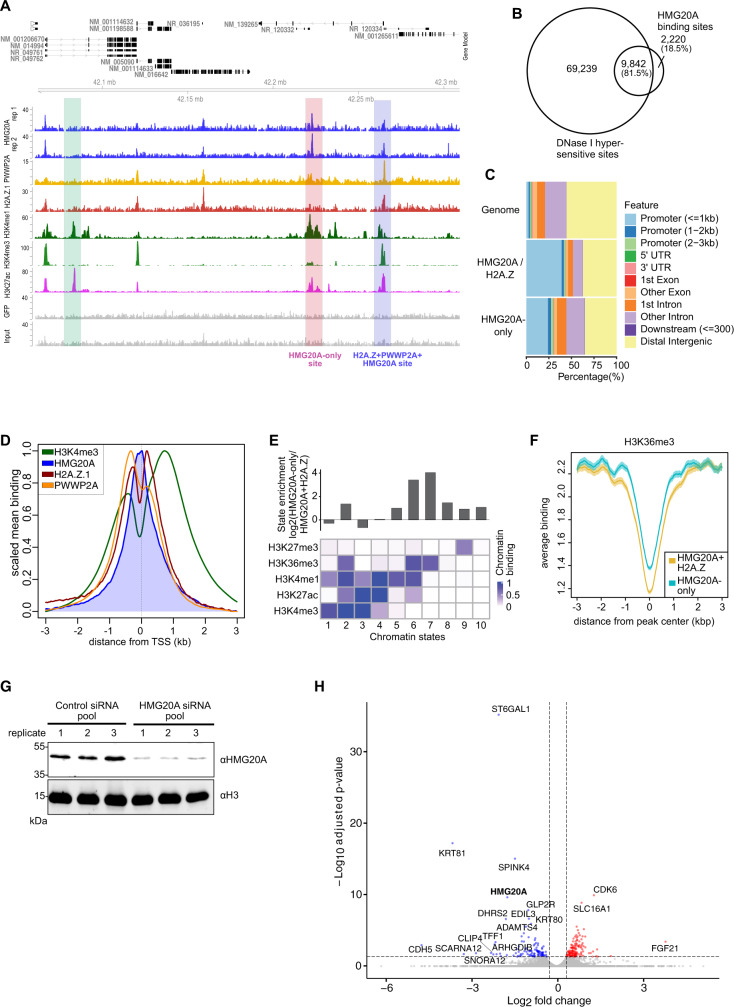
HMG20A localizes to distinct regulatory chromatin regions. **A** Genome browser snapshot of a representative region in human chromosome 15 displaying input (grey), GFP control (grey), H3K27ac, (purple), H3K4me3 (light green), H3K4me1 (dark green), GFP-H2A.Z.1 (red), GFP-PWWP2A (orange) and two replicates of GFP-HMG20A (blue) ChIP-seq signals (Pearson’s r = 0.88). Blue bar depicts HMG20A + H2A.Z.1 + PWWP2A-positive site, red bar depicts HMG20A-only site and green bar depicts a negative control site. **B** Venn diagram displaying numbers of HMG20A-bound sites and ENCODE published DNase I hypersensitive sites and their overlaps. **C** Enrichment plot representing genomic features of HMG20A + H2A.Z and HMG20A-only ChIP-seq sites. **D** Average binding profiles across transcriptional start sites of GFP-HMG20A (blue), -H2A.Z.1 (red), -PWWP2A (orange) and H3K4me3 (green) mean coverage signals at TSS of expressed genes. **E** ChromHMM^33,34^-based enrichment of chromatin states (defined by the specific combinatorial occurrence of five histone modifications) of GFP-HMG20A-only compared to HMG20A + H2A.Z-containing genomic regions. **F** Average binding plot of ENCODE H3K36me3-containing regions over HMG20A-only (yellow) and HMG20A + H2A.Z (blue) ChIP-seq sites. **G** Immunoblot of endogenous HMG20A upon siRNA-mediated depletion in HelaK cells (shown are three replicates). **H** Volcano plot of significantly deregulated (log2 fold change < −1, *p* < 0.05, calculated with Deseq2) mRNAs from two independent siRNA-mediated HMG20A depletion experiments analyzed by mRNA-seq. Red: upregulated transcripts, blue: downregulated transcripts. Source data for these figures are provided as a Source Data file.

Having revealed the DNA binding ability of HMG20A, we next mapped its genomic location by chromatin immunoprecipitation followed by high-throughput sequencing (ChIP-seq) (Fig. 3A), using HeLaK cells stably expressing GFP-HMG20A. We compared HMG20A sites with published ChIP-seq data for H2A.Z and PWWP2A^15,17^ and found two clusters: (i) HMG20A binding sites that overlapped strongly with H2A.Z/PWWP2A occupancy and (ii) HMG20A binding sites that overlapped weakly with H2A.Z/PWWP2A (HMG20A-only). (Fig. 3A, Supplementary Fig. 3A top). Of the approximately 12,000 HMG20A sites, around 70% overlapped with H2A.Z and/or PWWP2A regions (HMG20A + H2A.Z + PWWP2A sites), whereas around 30% showed reduced presence of H2A.Z and PWWP2A (we call them ‘HMG20A-only’ sites) (Supplementary Fig. 3A bottom). A subset of ChIP-seq enrichment sites were further validated by ChIP-qPCR (Supplementary Fig. 3B). All HMG20A-bound regions strongly overlapped with ENCODE available DNase I hypersensitive sites (Fig. 3B), suggesting that HMG20A resides at open chromatin regions.

In the presence of H2A.Z, HMG20A was found to be particularly enriched at regulatory regions, such as promoters and putative enhancers, while in the absence of H2A.Z HMG20A was additionally enriched at introns (Fig. 3C). The association of HMG20A with enhancer regions was independently confirmed by a strong correlation of HMG20A-bound regions with published STARR-seq sites^32^ (Supplementary Fig. 3C). A closer examination of HMG20A localization at promoters revealed enriched binding of HMG20A to nucleosome-depleted regions (NDRs) at transcriptional start sites (TSSs) that are surrounded by PWWP2A-bound H2A.Z-containing +1 and −1 nucleosomes (Fig. 3D). It is also notable that for all HMG20A binding sites (regardless of the presence or absence of H2A.Z), it was only full-length HMG20A, but neither the N-terminal nor the C-terminal region alone, that was able to pull down chromatin (Supplementary Fig. 3B), indicating that both DNA and HMG20A-interacting proteins are required for efficient chromatin binding (see Fig. 2C, D).

To further characterize and distinguish HMG20A + H2A.Z and HMG20A-only sites across the genome, we used available ENCODE ChIP-seq data sets to define the presence of H3K4me3 as promoter, H3K4me1 as enhancer and H3K27ac as active regulatory marks. HMG20A-only sites are biasedly less marked by PWWP2A and H3K4me3, while being mildly more H3K4me1-associated (Supplementary Fig. 3D). To increase our confidence and to better characterize genomic HMG20A binding regions, we performed a more powerful comparison between our datasets and chromatin states defined by training a 10-state model on ENCODE using ChromHMM^33,34^ (Fig. 3E). Interestingly, when comparing HMG20A-only associated histone modification pattern to those found at HMG20A + H2A.Z sites, HMG20A-only sites were also enriched in H3K4me1 and H3K36me3 (enhancer within transcribed gene bodies) (Fig. 3E, F). These observations suggest that HMG20A is associated with two major chromatin contexts: i) HMG20A together with H2A.Z and PWWP2A at mainly promoters enriched in H3K4me3 and ii) HMG20A-only sites in addition at intronic enhancers enriched in H3K4me1 within H3K36me3-positive, i.e., transcribed genes.

Next, we used MEME^35^ to ask whether the HMG20A enriched sites contain a specific consensus sequence. We found an overrepresented AT-rich motif in HMG20A-only sites, which resembles the published HMG-box binding motif AGAACAAGAAA^36^ (Supplementary Fig. 3E). Interestingly, additional representative sequences such as the promoter-associated Fos/Jun, SP, and KLF motifs were also detected.

As HMG20A resides at regulatory regions, we next tested whether HMG20A controls gene expression. We successfully depleted HMG20A in HeLaK cells using a specific siRNA-pool (Fig. 3G) and performed mRNA-seq. Surprisingly, only few genes were found to be affected in their transcription profile upon HMG20A depletion, with only 81 genes being up- and 77 down-regulated (Fig. 3H, Supplementary Data 2). These data imply that in HeLaK cells, HMG20A is not majorly involved in the control of gene expression, a finding that is supported by the observation that genes associated with GFP-HMG20A bound regulatory regions do not correlate with gene expression levels (Supplementary Fig. 3F).

Taken together, our data in HeLaK cells show that HMG20A localizes to NDRs within the TSSs of H2A.Z- and PWWP2A-surrounded promoter nucleosomes as well as to H2A.Z-less intronic enhancer regions within transcribed genes all containing defined consensus sequences, but it does not regulate gene expression in this cell line.

### HMG20A is required for craniofacial and heart development in *Xenopus laevis*

Our data so far indicated that despite HMG20A being localized to regulatory chromatin regions, it does not play a major role in transcriptional regulation in HeLaK cells. We therefore asked, whether it might have any function(s) during early developmental processes. Hence, we turned to the African clawed frog *Xenopus laevis* as an animal model. The *Xenopus* Hmg20a homologue shows high conservation in both the HMG box and the coiled-coiled region (Supplementary Fig. 4A). Whole-mount RNA in situ hybridization of various developmental stages, as well as RT-qPCR analyses, detected endogenous *X. laevis hmg20a* mRNA at all time points (Supplementary Fig. 4B). Maternal expression of *hmg20a* is seen at early cleavage and blastula stages (Supplementary Fig. 4C–F); zygotic expression is high at gastrula stages, where *hmg20a* is ubiquitously expressed (Supplementary Fig. 4G). At neurula stages, *hmg20a* is detected in neural folds and cranial NCCs (Supplementary Fig. 4H–J), followed by expression in migratory cranial NCCs, brain and eyes at tadpole stages (Supplementary Fig. 4K–R).

**Fig. 4:**
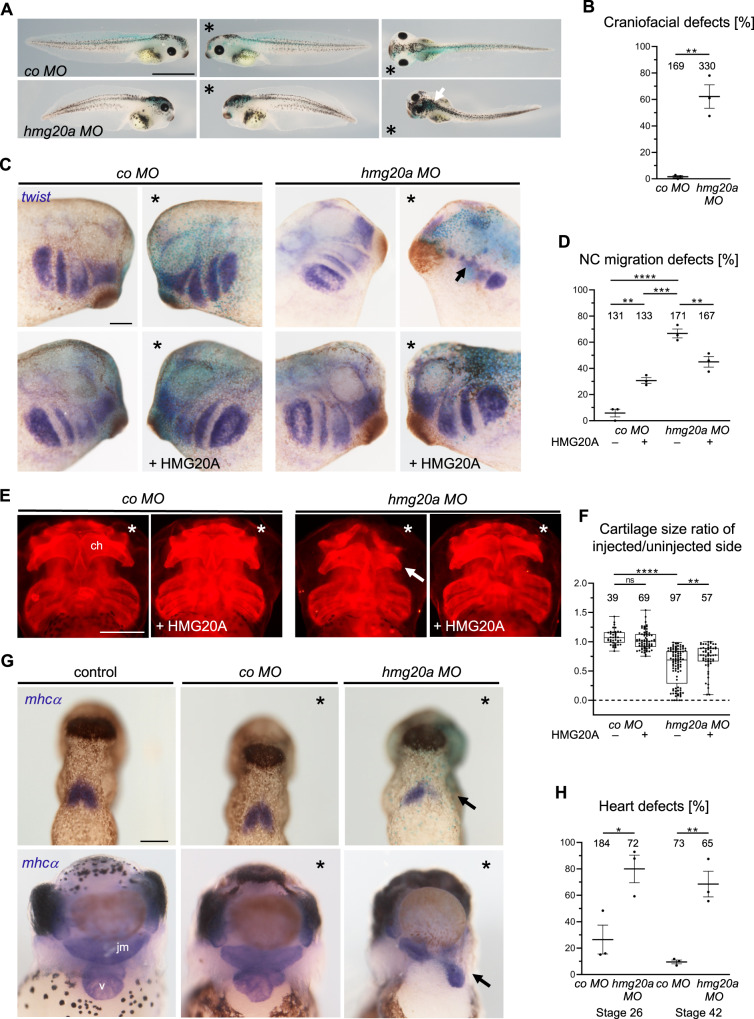
HMG20A depletion leads to craniofacial and heart malformations in frog. **A** Loss of function of Hmg20a leads to craniofacial and pigmentation defects in *Xenopus* tadpoles. *Marks the injected side, white arrow marks pigmentation defects. Scale bar = 1 mm. **B** Mean percentage of craniofacial defects of three independent experiments ± s.e.m. Number of embryos are indicated for each column. **p = 0.0024 (two-tailed unpaired Student’s t-test). **C** Hmg20 loss-of-function NC migration defects can partially be rescued by co-injection of human HMG20A DNA. * Marks the injected side (blue: lacZ staining, purple: *twis*t staining), arrow indicates cranial NC migration defect. Scale bar = 1 mm. **D** NC migration defects of three independent experiments injected and analysed as shown in (C). Data are presented as mean ± s.e.m., **p (left) = 0.0026, ***p = 0.0002 ****p = 0.0001, **p (right) = 0.006 (one-way ANOVA, Tukey’s multiple comparisons test). **E** Hmg20a-depleted *Xenopus* tadpole embryos show defects in cartilage formation (arrow). For rescue experiments, human *HMG20A* DNA was co-injected, *marks the injected side. Scale bar = 500 µm. **F** Box and whiskers plots summarize cartilage defects of at least three independent experiments analysed as in (E). Number of embryos (n, above each bar) and median are indicated. The box extends from 25th to 75th percentile, with whiskers from minimum to maximum. **p = 0.0013, ****p = 0.0001, ns.: not significant (one-way ANOVA, Tukey’s multiple comparisons test). **G** Hmg20a loss-of-function causes heart defects. Top: *mhcα* in situ hybridization reveals defects in the formation of the first heart field (arrow) at stage 26. Bottom: At stage 42, the three-chambered heart structure consisting of two atria (a) and a ventricle (v) is disturbed; the malformed heart is displaced towards the injected side (arrow). The jaw muscle (jm), which is also marked by *mhcα,* is also reduced. Scale bar = 1 mm. **H** Graph summarizing three independent experiments as shown in (**G**), data are presented as mean ± s.e.m. *p = 0.0242, **p = 0.0038 (two-tailed unpaired Student’s t-test). Source data for these figures are provided as a Source Data file.

To investigate the developmental role of *X. laevis* Hmg20a, we performed loss-of-function analyses. A translation blocking antisense Morpholino oligonucleotide (MO) targeted against the *hmg20a* RNA (*hmg20a MO*) or a control MO (*co MO*) was injected, in combination with *lacZ* RNA as a lineage tracer into one blastomere at the two-cell stage. At tadpole stages, morphants showed craniofacial and eye defects as well as hyperpigmentation on the injected side (Fig. 4A, B), for the most part indicative of defects in NCC development. To further analyse NCC migration, we performed in situ hybridization of *hmg20a MO-*injected embryos and controls using the NCC marker *twist*. Indeed, we observed a reduction of twist-positive migratory NCCs on the *hmg20a MO-*injected side (Fig. 4C, D), which could be partially rescued by co-injection of human *HMG20A* cDNA (Fig. 4C, D). As NCCs contribute to the formation of cranial cartilage, we performed collagen II immunostaining to visualize the cartilage of morphant and control embryos. Consistent with a function of Hmg20a in NCC migration, we observed a reduction of cartilage structures on the *hmg20a MO*-injected side compared to embryos injected with the *co MO* (Fig. 4E, F). Again, these defects were partially rescued by co-injection of human *HMG20A* cDNA (Fig. 4E, F). Interestingly, the expression pattern and NCC-specific loss-of-function defects of *Xenopus hmg20a* are highly reminiscent of those previously observed for *Xenopus* Pwwp2a^15^, suggesting a partial functional overlap between these associated proteins.

*Hmg20a*-depleted tadpoles also showed defects in heart morphology (Fig. 4G, H), We traced the expression of the cardiac differentiation marker *myosin heavy chain alpha* (*mhcα*) by in situ RNA hybridization. As shown in the top panel of Fig. 4G, *hmg20a MO* injected embryos showed a reduction of *mhcα* in the first heart field compared to controls in earlier tadpole stages. Later at stage 42, control and *co MO*-injected embryos showed the typical heart structure consisting of two atria and one ventricle (Fig. 4G bottom, H). However, this three-chambered heart structure could not be distinguished in *hmg20a MO* injected embryos. Furthermore, the malformed hearts were displaced toward the side of the injection.

Taken together, our data suggest that *Xenopus* Hmg20a plays an essential role in craniofacial and heart morphogenesis.

### Hmg20a drives neural crest cell and cardiomyocyte differentiation of mESCs

**Fig. 5:**
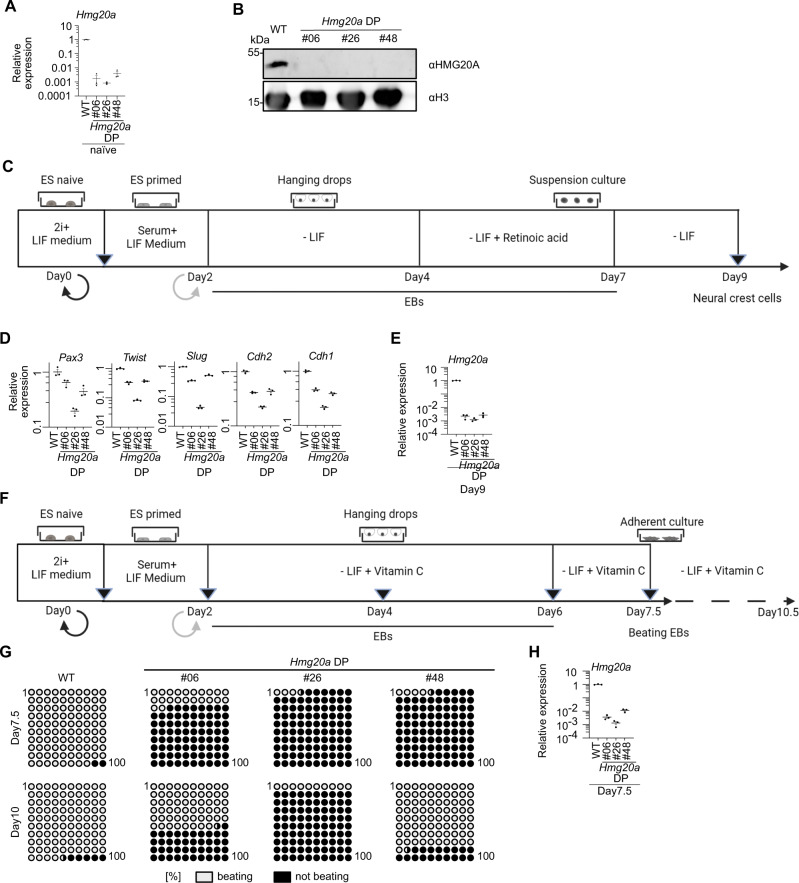
HMG20A is essential for cardiomyocyte and neural crest differentiation in mESCs. **A** Validation of three *Hmg20a* DP clones by RT-qPCR. Shown is *Hmg20a* expression normalized to *Hprt*. Data is presented as mean ± SEM of three technical replicates. **B** Immunoblot analyses of endogenous HMG20A protein of extracts from WT and three *Hmg20a* DP mESC clones. H3 served as loading control (shown is one representative blot of two consistent replicates). **C** mESC neural crest cell (NCC) differentiation scheme. Created with BioRender.com. **D** RT-qPCR of neural crest and EMT marker genes in WT and three individual *Hmg20a* DP clones at Day9 of neural crest differentiation protocol normalized to *Hprt, 18* *S RNA and Gapdh* expression. Data is presented as mean ± SEM of three technical replicates. **E** RT-qPCR of *Hmg20a* mRNA in WT and three individual *Hmg20a* DP clones at Day9 of neural crest differentiation protocol. Expression was normalized to *Hprt, 18* *S RNA and Gapdh* expression. Data is presented as mean ± SEM of three technical replicates. **F** mESC cardiomyocyte (CM) differentiation scheme. Created with BioRender.com. **G** Depiction of percent beating (gray) or non-beating (black) WT or three individual *Hmg20a* DP EBs at Day7 (Top) or Day10 (bottom) of the cardiomyocyte differentiation procedure (see **G**). Ability to form contracting cardiomyocytes on Day7.5 is significantly reduced in all *Hmg20a* DP clones (Fischer’s exact test, two-sided, *p* = 3.1213974265e-087 (#06), *p* = 1.1876278705e-122 (#26), *p* = 2.0898552635e-091 (#48)). **H** RT-qPCR of *Hmg20a* mRNA in WT and three individual *Hmg20a* DP clones at Day7.5 of CM protocol. Expression was normalized to *Hprt, 18* *S RNA and Gapdh* expression. Data is presented as mean ± SEM of three technical replicates. Source data for these figures are provided as a Source Data file.

Since Hmg20a depletion in *X. laevis* resulted in severe craniofacial and heart defects (see Fig. 4), we asked whether the function of HMG20A is evolutionarily conserved and whether such defects could be recapitulated in the mammalian system. Hence, we generated viable *Hmg20a*-depleted (DP) mESCs by introducing a triple-terminator sequence behind the first ATG of the *Hmg20a* gene using a CRISPR/Cas9-based approach (Fig. 5A, B, Supplementary Fig. 5A). We successfully differentiated WT mESCs into NCCs^37^ (Fig. 5C) as assessed by RT-qPCR of several NCC and/or EMT marker genes (Supplementary Fig. 5B), mimicking the developmental process related to the formation of craniofacial structures. Compared to WT cells, *Hmg20a* DP mESCs inefficiently differentiated into NCCs as assessed by RT-qPCR analysis using the same marker genes (Fig. 5D). In addition, *Hmg20a* DP embryoid bodies (EBs) showed a slightly reduced migration capacity (Supplementary Fig. 5C), with the severity of the phenotype correlating to the amount of residual *Hmg20a* mRNA expression levels (Fig. 5E). These data suggest that HMG20A is important for proper NCC migration and differentiation.

Due to the observed reduction of *mhcα* expression in *Hmg20a*-depleted *X. laevis*, we next focused on the differentiation of WT and *Hmg20a* DP mESCs into beating CMs^38^ (Fig. 5F, Supplementary Fig. 5D). As a result, differentiation of mESCs to CMs was also compromised after depletion of HMG20A, which was manifested by significantly smaller EBs at the intermediate stage (Supplementary Fig. 5E, F). More importantly, *Hmg20a* DP mESCs did not produce beating CMs in a timely manner on differentiation Day7.5 compared to WT cells (Fig. 5G top, Supplementary Movies 1-4). Yet, some more *Hmg20a* DP CMs started beating from Day10 on (Fig. 5G bottom), suggesting that loss of HMG20A does not cause a complete stop of cardiomyocyte differentiation but rather results in a severe delay. Once again, the severity of the observed beating phenotype was directly correlated to residual *Hmg20a* mRNA expression levels (Fig. 5H), suggesting that the Hmg20a mRNA/protein dosage is important for its functional output.

In summary, our in vitro mESC model phenocopies the defects observed in vivo in *Xenopus*, corroborating our findings that HMG20A is functionally conserved and required for proper differentiation to NCCs and beating CMs.

### HMG20A regulates genes involved in NCC and cardiomyocyte differentiation

**Fig. 6:**
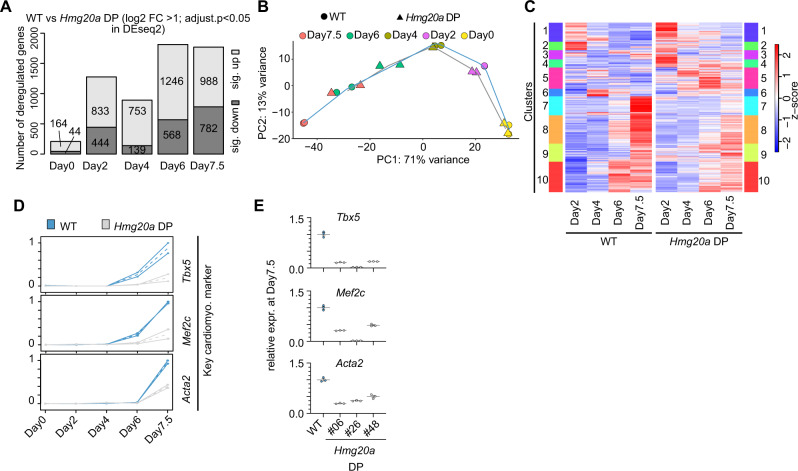
HMG20A regulates cardiomyocyte transcription programs. **A** Stacked Bar plot of numbers of significantly up (log2 fold change >1) and down (log2 fold change < −1) regulated genes (adjusted *p*-value < 0.05) during indicated time points of cardiomyocyte differentiation of WT and *Hmg20a* DP clone #26 mESCs as identified by mRNA-seq. **B** Principle component Analysis (PCA) of RNA-seq data of two replicates of WT (circle) and *Hmg20a* DP clone#26 (triangle) at Day0 (yellow), Day2 (magenta), Day4 (olive), Day6 (green) and Day7.5 (red) differentiation time points (see Fig. 5F for cardiomyocyte differentiation scheme). **C** Heat map showing the z-scaled expression values from all significant deregulated genes comparing the differentiation steps (Day 2 vs. 4; 4 vs. 6; 6 vs. 7.5). Genes are clustered according to the Euclidean distance by an unsupervised agglomerative hierarchical approach. Shown are the mean z-scales of two replicates for each day for WT (left panel) or *Hmg20a* DP (right panel) cells. **D** Line plots showing the min-max normalized mRNA-seq expression values for known cardiomyocyte marker genes at various differentiation time points. Plotted are mean expression values (dotted line) and the standard deviation (continuous line) for WT (blue) or *Hmg20a* DP (grey) cells. **E** RT-qPCR of cardiomyocyte marker genes in WT and three individual *Hmg20a* DP clones at Day7.5 of cardiomyocyte differentiation protocol normalized to *Hprt, 18* *S RNA and Gapdh* expression. Data is presented as mean ± SEM of three technical replicates. Source data for these figures are provided as a Source Data file.

To better understand the molecular cause of the observed phenotypes, we examined whether the transcriptomes of *Hmg20a* WT versus DP cells changed at different time points during mESC differentiation to CMs. mRNA-seq experiments were performed on naïve (Day0), primed (Day2), two EB stages (Days4 and 6) and CMs (Day7.5) of WT and *Hmg20a* DP clone #26 cells (see Fig. 5C). We chose this clone as it showed the least residual *Hmg20a* mRNA expression levels as well as the most severe phenotypes (see Fig. 5). Over time, an increasing number of deregulated genes were detected in *Hmg20a* DP cells (Fig. 6A, Supplementary Data 3). Principal Component Analysis (PCA) revealed a striking stage-dependent gene expression trajectory (Fig. 6B). *Hmg20a* DP Day7.5 cells showed a transcriptome profile comparable to that of Day6 WT cells, mirroring also on the transcriptome level the severe developmental delay observed during differentiation of *Hmg20a* DP mESCs into CMs. To further analyze these differences, we examined changes in gene activity in WT cells, dividing them into 10 clusters according to their behaviour over time and compared them to the *Hmg20a* DP data (Fig. 6C). Several clusters showed critical functions in i.e., EMT and heart process/morphogenesis (Supplementary Fig. 6). Examples of key cardiomyocyte marker expression changes during CM differentiation are shown in Fig. 6D. Although a slight clonal effect was observed between the three *Hmg20a* DP clones, expression changes were validated by RT-qPCR for core cardiac TFs (Mef2c, Tbx5)^39^ and the myofibroblast/cardiomyocyte marker Acta2^40^ (Fig. 6E), thus reinforcing our observation of HMG20A’s role in regulating CM transcription programs.

Together, these results indicate that HMG20A is a key player in the regulation of lineage-specific differentiation transcription programmes.

### HMG20A is found at open chromatin regions in mESCs and affects chromatin accessibility

**Fig. 7:**
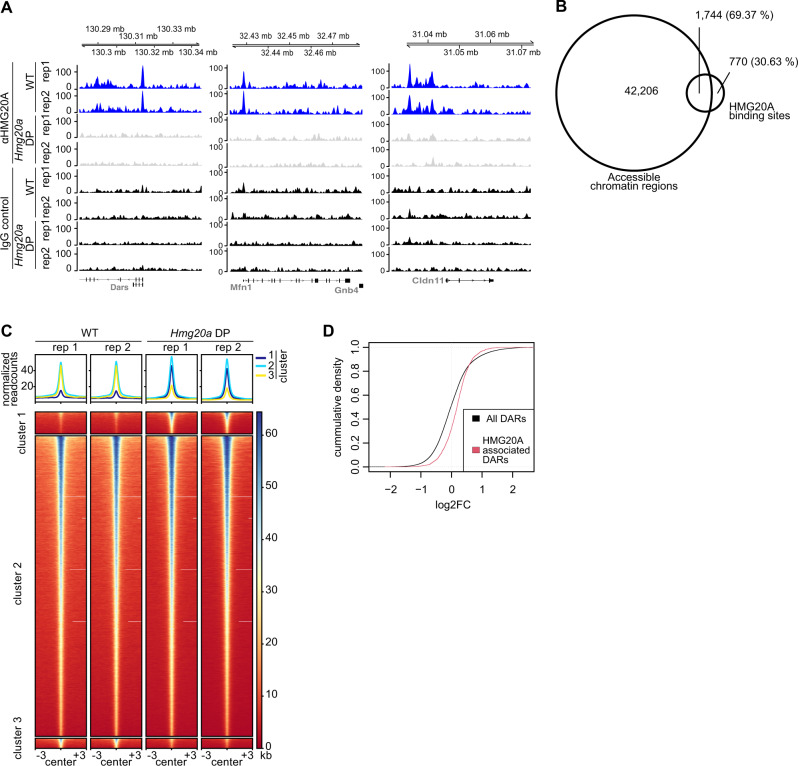
HMG20A is enriched at open chromatin regions and affects chromatin accessibility in mESCs. **A** Genome browser snapshots of representative HMG20A binding regions, as identified by CUT&RUN-seq of primed stage WT (blue) or *Hmg20a* DP clone #26 (light gray, negative control) mESCs. IgG antibody was used as negative control (black). Shown are two independent replicates (rep). **B** Venn diagram depicting overlay of CUT&RUN identified HMG20A binding sites with ATAC-seq identified accessible chromatin regions. **C** Density heatmap of ATAC-seq sites (two replicates) that become more open (cluster 1), remain unaffected (cluster 2) or become more closed (cluster 3) upon *Hmg20a* depletion. **D** Cumulative density plot showing the distribution of the observed changes in chromatin accessibility (shown as log2FC (DP/ET)) for all ATAC-seq signals (black) and for those ATAC-seq signals overlapping with HMG20A (red). DARs: differentially accessibly regions.

Having demonstrated HMG20A’s participation in regulating the expression of genes involved in NCC and CM differentiation, we wondered what HMG20A’s direct genomic targets are. We investigated the genome-wide localization of endogenous HMG20A by performing CUT&RUN in WT and *Hmg20a* DP mESCs at the primed stage, as this resembles an important joint point just before the experimental separation of both NCC or CM differentiation protocols. We identified a total of 2,545 bona fide HMG20A-binding peaks, corresponding to 2,094 genes (Fig. 7A, Supplementary Fig. 7A). Peaks were generally characterized as specific by the presence of signals in WT and their absence in the *Hmg20a* DP clone #26.

Next, we determined whether HMG20A binding sites correlate with gene activity. In contrast to GFP-HMG20A in HeLaK cells, genes close to HMG20A CUT&RUN binding-peaks in mESCs were slightly more strongly expressed the more HMG20A was bound to their corresponding regulatory regions (Supplementary Fig. 7B). As gene expression levels and open chromatin states are closely related, we monitored HMG20A-dependent chromatin accessibility by performing ATAC-seq in primed WT and *Hmg20a* DP mESCs. Similar to the observed significant overlap between GFP-HMG20A binding regions and DNase I hypersensitive sites in HeLaK cells (see Fig. 3B), we found approximately 70% of HMG20A binding-peaks within chromatin accessible sites (Fig. 7B). This finding suggests that, also in mESCs, most of HMG20A protein binds to open chromatin structures, possibly regulatory regions. While the majority of chromatin did not show any changes in accessibility upon HMG20A loss (Fig. 7C, cluster 2), we observed several differentially affected regions (DARs), with cluster 1 DARs gaining while cluster 3 DARs loosing accessibility (Fig. 7C). These observed chromatin structure changes directly correlated with changes in transcription levels of corresponding genes. Genes associated with increased chromatin accessibility showed an upregulation in their gene expression profile (Supplementary Fig. 7C, left), whereas genes associated with decreased accessibility showed reduced expression (Supplementary Fig. 7C, right). Interestingly, a majority of HMG20A-associated DARs showed an increase in accessibility (Fig. 7D), suggesting that HMG20A is mainly implicated in the ‘down-tuning’ of gene expression within HMG20A-DARs.

In summary, HMG20A is associated with open chromatin regions in mESCs and its loss results in changes in chromatin accessibility, mainly chromatin opening.

### HMG20A binds to regulatory regions controlling embryo morphogenesis and chromatin organization in mESCs

**Fig. 8:**
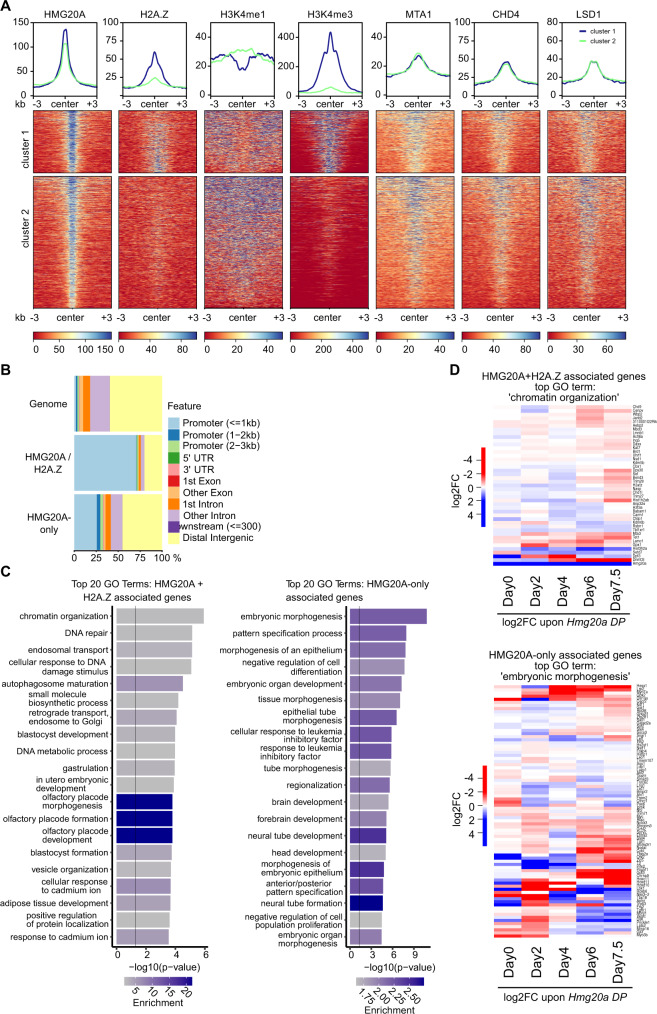
HMG20A localizes to promoters and enhancers that control genes involved in morphogenesis and chromatin organization in mESCs. **A** Density heatmap of HMG20A binding sites detected in CUT&RUN (see Fig. 7B) compared to publically available H2A.Z, H3K4me1, H3K4me3, MTA1, CHD4 and LSD1/KDM1A ChIP-seq data from mESCs computationally (k-means) separated into two clusters: cluster 1 (blue line top): HMG20A + H2A.Z and cluster 2 (green line top): HMG20A-only sites. Color intensity represents normalized and globally scaled tag counts. **B** Enrichment plot representing genomic features of HMG20A + H2A.Z and HMG20A-only CUT&RUN sites. **C** Top 20 GO term analysis of genes associated with CUT&RUN clusters 1 and 2 peaks identified in Fig. 8A, B. Calculated by metascape.org^69^. **D** Heatmaps of expression changes (DP versus WT) of genes within top GO terms ‘chromatin organization’ (top) or ‘morphogenesis’ (bottom) (see Fig. 8C) at the different differentiation steps.

To better characterize the identified HMG20A binding sites in mESCs by CUT&RUN, we performed comparative analyses of available ENCODE H2A.Z, H3K4me1 and H3K4me3 ChIP-seq data (Fig. 8A). We identified two HMG20A binding-peak clusters: i) associated with H2A.Z (HMG20A + H2A.Z) and enriched in H3K4me3 (promoter mark), and ii) without H2A.Z (HMG20A-only) and enriched in H3K4me1 (enhancer mark). HMG20A + H2A.Z binding-peaks were located mainly in promoter regions, while HMG20A-only binding-peaks showed an additional enrichment at introns, similar to our observations in HeLaK cells (Figs. 8B and 3B). Sequence analyses of the distinct HMG20A binding-peak regions revealed a strong enrichment of SP and A-rich motifs, too (Supplementary Fig. 8). Therefore, both the genomic distribution of HMG20A at regulatory regions and its bound DNA consensus motifs are strongly conserved between human HeLaK and mouse ES cells.

Next, we wondered, whether HMG20A peaks overlapped with binding sites for NuRD and BHC complexes in mESCs, as these proteins were found to interact with GFP-HMG20A in HeLaK cells (see Fig. 1C, D). Using published LSD1/KDM1A (BHC complex member) as well as MTA1 and CHD4 (NuRD complex members) ChIP-seq data from mESCs, we indeed found these proteins at HMG20A binding-peaks, regardless of H2A.Z’s presence or absence (Fig. 8A), supporting an (indirect) chromatin association between these factors and HMG20A in mESCs.

To identify the biological functions of those genes controlled by HMG20A + H2A.Z or HMG20A-only regulatory regions, we determined their GO terms. We found HMG20A-only associated genes to be highly enriched in developmental processes involved in ‘embryonic morphogenesis’, while HMG20A + H2A.Z-linked genes were associated with more basic DNA-regulating processes, such as ‘chromatin organization’ and other cellular response processes (Fig. 8C). Underscoring the functional relevance of the assigned GO terms, we observed that many of the genes associated with these GO terms were deregulated in *Hmg20a* DP primed cells (Fig. 8D).

Taken together, these findings strongly support the notion that HMG20A is involved in embryogenesis and stem cell differentiation, probably by the regulation of genes involved in modulating chromatin structure and affecting specific developmental gene expression programs.

## Discussion

**Fig. 9:**
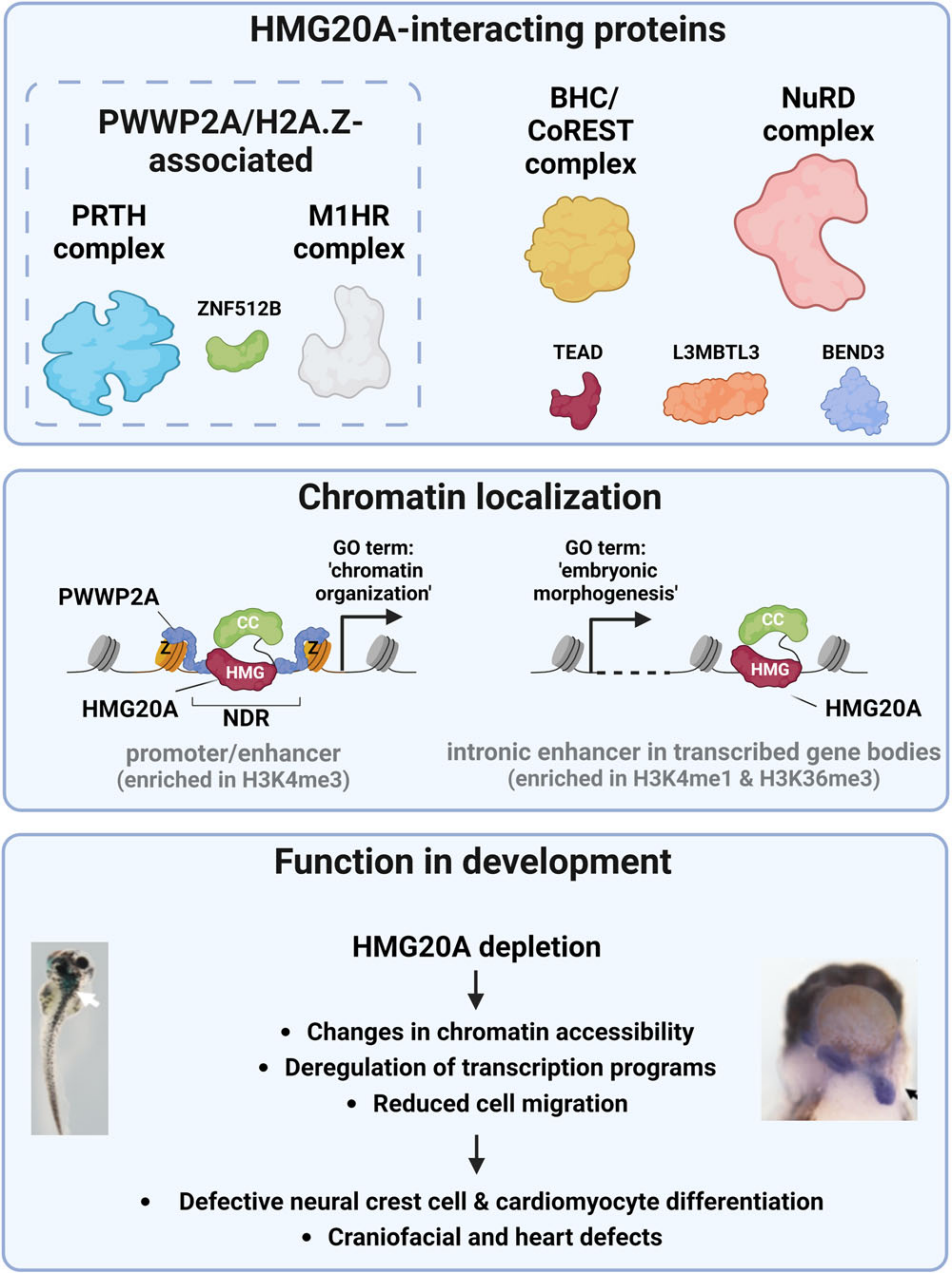
Model of HMG20A’s function in chromatin and transcriptional regulation during development. Top: HMG20A associates with H2A.Z- and PWWP2A-associated PRTH and M1HR complexes and ZNF512B as well as BHC/CoREST and NuRD complexes and TEAD and L3MBTL3 that are not part of H2A.Z or PWWP2A interactomes. Middle: HMG20A binds to two distinct chromatin regulatory elements: (1) Nucleosome depleted regions (NDR) at promoter sites that are surrounded by H2A.Z-containing nucleosomes and bound by PWWP2A and that are associated with genes involved in basic processes, such as ‘chromatin organization‘. (2) H2A.Z-lacking intronic enhancers within transcribed genes belonging to developmental processes, such as ‘embryonic morphology”. Bottom: Depletion of HMG20A in *Xenopus laevis* and mESCs leads to changes in chromatin accessibility, deregulation of transcription programs as well as migration defects. HMG20A depleted cells fail to properly differentiate into neural crest cells or cardiomyocytes in mESCs as well as head and heart in *Xenopus laevis*. Figure was created with BioRender.

Our work provides a deeper understanding of HMG20A’s function as transcriptional regulator during differentiation processes. Physically, by revealing the interactome of HMG20A we identified known interactors of the BHC/CoREST and PRTH complexes, as well as all subunits of the NuRD complex and other chromatin-modifying proteins (Fig. 9, top). We further characterized the functional domains of HMG20A responsible for NuRD interaction and DNA binding. Mechanistically, we revealed HMG20A’s conserved genomic distribution in human and mouse cells. HMG20A preferentially binds to open chromatin regions, in particular to NDRs surrounded by H2A.Z-nucleosomes as well as to enhancers within introns of transcriptional active genes that do not contain H2A.Z (Fig. 9, middle). Absence of HMG20A leads to major changes in chromatin accessibility resulting in more opened chromatin regions. Phenotypically, we uncovered a conserved function of HMG20A in NCC-linked head as well as CM-linked heart development (Fig. 9, bottom). During mESC differentiation, we find HMG20A to be bound to regulatory regions of genes whose functions are associated with processes such as ‘morphogenesis’ and ‘chromatin organization’ and which are deregulated when HMG20A is missing. Hence, HMG20A participates in the control of chromatin structure and transcription of genes that are crucial for cell migration and proper cell fate decision. In summary, HMG20A serves as a key modulator of faithful differentiation of stem cells into neural crest cells and cardiomyocytes by guiding transcription programs involved in lineage commitment.

### HMG20A associates with several chromatin-modifying complexes and binds to two distinct types of open regulatory chromatin regions

The binding partners of HMG20A we identified in HeLaK cells can be divided into two groups (Fig. 9, top): The first group comprises BHC/CoREST, a known HMG20A associated complex identified by us and others^28^, the complete NuRD complex, and several chromatin-modifying proteins. Members of this group are not part of the H2A.Z^15,21^ and PWWP2A^16^ interactomes. The second group comprises proteins that are also associated with H2A.Z and PWWP2A, namely members of the PRTH complex and the MTA1, HDAC and RBBP4/7 core components of the NuRD complex (M1HR). We observed that in contrast to PWWP2A, which exclusively interacts with the M1HR subcomplex of NuRD, the PWWP2A interactor HMG20A is able to pull-down components of both deacetylase and remodelling subcomplexes of NuRD. Our ChIP-seq and CUT&RUN findings in human and mouse cells show that HMG20A binds to two distinct types of chromatin loci. On the one hand, HMG20A is associated to H2A.Z-cobound promoters. On the other hand, it is found, additionally, at intronic enhancers in the absence of H2A.Z (Fig. 9, middle). Thus, it is tempting to speculate about two working contexts (at least in HeLaK cells) for HMG20A, one in which HMG20A associates with PHF14 of the PRTH complex^31^ and the M1HR complex at H2A.Z/PWWP2A^18^-cobound promoters, and a second at intronic enhancer sites where it interacts with the complete NuRD and the BHC/CoREST complexes and possibly also TEAD, L3MBTL3 and BEND3, as those are also not found in H2A.Z or PWWP2A interactomes. However, HMG20A appears to co-localize with MTA1, CHD4 and LSD1 at H2A.Z-positive regulatory regions in mESCs, suggesting that such a clear distinction cannot be made in all cell types.

Given the highly similar motifs found in both HMG20A-only and HMG20A + H2A.Z binding sites and the failure of the HMG box-containing N-terminus to pull-down chromatin, it is unlikely that DNA sequence alone is the primary factor determining HMG20A’s chromatin targeting. However, future studies will be needed to evaluate whether other interacting proteins, such as BHC/CoREST, NuRD subunits, PRTH members, PWWP2A, H2A.Z and others, help to target/attach HMG20A to distinct regulatory regions, maybe even in combination with specific DNA sequences and structure^31^.

### HMG20A controls head and heart development in *Xenopus* and NCC and CM differentiation programs in mESCs

We found that HMG20A is required for proper craniofacial and heart formation during embryonic development. The observed defect in craniofacial formation is likely related to reduced cell migration and early differentiation problems of HMG20A-depleted NCCs. NCCs are defined as a multipotent cell population and also as a fourth germ layer in the developing embryo that contributes to the formation of a wide range of tissues, including craniofacial cartilage, heart and pigment cells^41^. Therefore, compromised differentiation to NCCs after HMG20A depletion could also partially explain the observed heart defects. Additionally, we have also observed hyperpigmentation in *Hmg20a*-depleted tadpoles, which could be due to a biased differentiation of NCCs to pigment cells of the skin at the expense of other cell types/tissues. Furthermore, our finding complements a previous report implicating HMG20A in neuronal development and skeletal muscle differentiation^24,42^. In order to migrate, NCCs have to undergo an epithelial to mesenchymal transition (EMT). Here, we demonstrate that HMG20A is required for EMT and cell migration in mouse NCCs, which at least partially explains our observed phenotypes. In line with our finding, a previous study reported that HMG20A is required for SNAI1-mediated EMT by replacing HMG20B, while its loss induces reversion of the EMT signaling program^28^. Also, another report which was published during the revision process of this manuscript showed that loss of HMG20A impairs TGFβ-triggered EMT^31^.

### Hypothesis on how HMG20A and its associated complexes regulate transcription programs during differentiation

But how can HMG20A mechanistically contribute to these processes? We speculate that it is involved in the regulation of early transcription programs. We find HMG20A at open chromatin sites with its binding strength being positively correlated with transcription levels. On the other hand, removal of HMG20A results in chromatin accessibility changes with more regions opening than closing (Fig. 9 bottom). These, on the first sight, contradictory observations could be explained with HMG20A acting as rheostat and thereby fine-tuning the expression of highly transcribed genes. Such a function has been previously described for the NuRD complex^43^ and RCOR1 of the BHC/CoREST complex^44^. It is therefore tempting to speculate that HMG20A – among other functional outputs – works together with these complexes to dampen transcription of genes during differentiation. This hypothesis is also in line with the finding that HMG20A depletion leads to more upregulated genes at early mESC differentiation time points.

Interestingly, GO terms of Day2 deregulated genes in HMG20A depleted primed mESCs reveal affected Ras and Rho signal transduction pathways. These have been shown to be involved in cell migration and EMT^45,46^ and thereby contributing to embryo development. As also genes associated with HMG20A-only bound regulatory regions belong to the GO term ‘embryonic morphogenesis’, we speculate that HMG20A (possibly together with NuRD and BHC/CoREST complexes) fine-tunes expression of those important EMT genes leading to severe NCC and CM defects.

Additionally, BHC/CoREST with LSD1 are also well-known factors involved in neural stem cell biology and neural development^47^. Previous studies suggest that the interaction of HMG20A with the BHC/CoREST complex plays an important role in the initiation of neuronal differentiation^24,48,49^. Recently, the CHD4/NuRD complex was reported to regulate neural differentiation of ESCs^50^. Since neuronal differentiation is associated with NCCs, either BHC/CoREST or NuRD complexes could function, together with HMG20A, during NCC differentiation. On the other hand, LSD1 has been shown to play a role in heart development via its interaction with binding partners (e.g., BHC/CoREST) and enzymatic activities^51^. The CHD4/NuRD complex has also recently been demonstrated to directly control cardiac sarcomere formation in the developing heart in mice^52^. Together, these studies indicate the potential involvement of HMG20A/LSD1-CoREST and/or HMG20A/CHD4-NuRD complexes in cardiac development.

TEAD1, which is one of the core cardiac transcription factors in heart development^39^ was also repeatedly detected in our HMG20A interactome data sets and recently described to bind to HMG20A^31^. Apart from these interacting partners that we identified, HMG20A was also shown to bind to Ca2 + /S100A6, a protein that contributes to cellular calcium signalling^53^. Very interestingly, we found that beating of *Hmg20a* DP cardiomyocytes was abolished or delayed. This could be due to the lack of interaction with Ca^2+^/S100A6, which leads to the loss of calcium signalling. In the future, depletion of individual HMG20A-associated proteins in combination with genome-wide profiling of their localization in *Hmg20A* DP mESCs will be required to further elucidate the role of HMG20A and its binding partners in specific lineage commitment.

In conclusion, our findings implicate HMG20A as part of the H2A.Z/PWWP2A/NuRD-axis residing within distinct open regulatory regions, thus acting as a key modulator in orchestrating specific transcription programs to ensure proper lineage differentiation.

## Methods

### Cell culture

HeLa Kyoto (HeLaK) cells were grown in Dulbecco’s modified Eagle’s medium (DMEM, Gibco) supplemented with 10% fetal calf serum (FCS; Gibco) and 1% penicillin/streptomycin (37 °C, 5% CO_2_) and routinely tested with PCR for mycoplasma contamination. mESCs were cultivated in 2i + LIF condition as previously described^54^. Specifically, mESCs were cultured on 0.1%-gelatin-coated 6-well plates coated with 0.1% gelatin in N2B27 medium supplemented with 50 μM β-mercaptoethanol, 2 mM L-glutamine, 0.1% Sodium bicarbonate, 0.11% bovine serum albumin fraction V, 1000 units/ml recombinant mouse leukemia inhibitory factor (LIF), 1 µM PD0325901 (MEK inhibitor) and 3 µM CHIR99021 (GSK3 inhibitor). Every two days, cells were passed at a density of 1.4 × 10^5^ cells/well. Embryoid body (EB)-mediated differentiation into cardiomyocytes was performed as previously described^38^. Briefly, on differentiation Day0, naïve mESCs (2i+LIF) were adapted to primed state in differentiation media (DMEM, Gibco) supplemented with 10% FCS, 2 mM L-glutamine, 1% nonessential amino acids, 0.1 mM β-mercaptoethanol, 1000 U/mL leukemia inhibitory factor (LIF) for 2 days. Hanging drops containing 1000 cells were prepared in 25 µl of differentiation medium (without LIF) supplemented with 50 µg/ml vitamin C. On differentiation Day6 (4 days in suspension), each droplet containing one EB was carefully transferred to a 24-well plate coated with 0.1% gelatin. Beating cardiomyocytes were observed starting from differentiation Day7. NCC differentiation was carried out based on the protocol previously described^37^. Briefly, on differentiation Day0, naïve mESCs (2i+LIF) were adapted to primed state in above mentioned differentiation media for 2 days. Hanging drops containing 1000 cells were prepared in 25 µl of differentiation media (without LIF) for 2 days. Then, EBs were pooled and cultured in suspension in differentiation media (without LIF) supplemented with 0.1 µM retinoic acid (RA) for 3 days, followed by 2 days without RA. On differentiation Day9, the formed EBs containing NCCs were collected for downstream analysis. Transfections were performed using FuGENE® HD Transfection Reagent according to the manufacturer’s instructions (Promega). 24 h after transfection, puromycin (0.25 µg/ml) was used to select mESCs for at least 10 days. Finally, mCherry-positive mESC colonies were picked for further culture and characterization by RT-qPCR and western blotting, respectively. Sf9 cells were cultured in Sf-900TM II SFM medium (Gibco) and maintained at 27 °C and 90 rpm.

### Plasmids

To generate human HMG20A (truncation) fusion proteins, total RNA of HelaK cells was purified using RNeasy-Kit (QIAGEN) and reverse transcribed using Transcriptor First Strand cDNA Synthesis Kit (Roche). cDNA was amplified by Q5-DNA polymerase. (New England Biolabs) and cloned into pIRESneo-GFP^4^ and pFASTBAC1 (Invitrogen) vectors (FLAG was introduced by PCR primer) vectors. To generate in situ hybridization probes against *hmg20a.L* RNA, *Hmg20a* cDNA from *X. laevis* embryos was amplified and cloned into pcDNA3.0. To generate *Hmg20a* DP mESC cell lines, CRISPR/Cas9 technology was used. Briefly, sgRNAs were designed to target the start codon (ATG) and the first intron of *Hmg20a* using the online tool (http://crispor.tefor.net), synthesized by Integrated DNA Technology (IDT), and cloned into the vector pX461 (Addgene). Donor pUC19-based vectors containing a selectable marker, either mCherry or puromycin resistance gene, and mammalian transcriptional triple terminators *bGH* + *hGH* + *SV40* (synthesized by GENEWIZ) flanked by homology arms, generated by PCR using Q5-DNA polymerase from mESC genomic DNA obtained using the QIAamp DNA Mini Kit (QIAGEN), were constructed by HiFi DNA Assembly (New England Biolabs). All relevant sgRNA sequences and primers are listed in Supplementary Data 4.

To perform NuRD binding immunoprecipitations, pcDNA3.1 constructs were prepared that encode full-length genes for human CHD4 (UniProt ID: Q14839), RBBP4 (UniProt ID: Q09028), MTA1 (UniProt ID: Q13330), GATAD2A (UniProt ID: Q86YP4), MBD2 (UniProt ID: Q9UBB5)^27^, MBD3 (UniProt ID: O95983), and MTA2 (UniProt ID: O94776), with N-terminal FLAG or HA tags. The exception was full-length HDAC1 (UniProt ID: Q13547) which had the FLAG tag at the C-terminal end^27^. We also used pcDNA3.1 constructs coding for flag-tagged human CHD4 HMG box (residues 1–355), DNA translocase (residues 343–1230), and C-terminal domains (residues 1230–1912).

### siRNA transfections

To deplete HMG20A in HeLaK, 2 × 10^5^ or 3 × 10^6^ cells were transfected with 20 pmol of ON-TARGETplus Human HMG20A (10363) siRNA-SMART pool (Dharmacon) using Oligofectamine™ according to the manufacturer’s instructions (Invitrogen). Cells were cultured for 3 days before being harvested for subsequent experiments.

### Antibodies

All antibodies used are listed in Supplementary Data 4.

### Fluorescence microscopy of HMG20A fusion proteins

1×10^5^ HelaK cells expressing GFP, GFP-HMG20A, GFP-HMG, GFP-CC were seeded on glass plates and cultured overnight. The next day, cells were washed with PBS and fixed for 10 min in 1% formaldehyde in PBS. After washing, fixed cells were permeabilized with 1% bovine serum albumin (BSA) in PBS premixed with 0.1% Triton-X-100 for 30 min. Endogenous HMG20A protein was stained by stepwise incubation with primary and then secondary Alexa Fluor–conjugated antibody for 45 min. To visualize DNA, cells were treated with 10 µg/ml Hoechst. Coverslips were mounted in Fluoromount-G mounting medium (SouthernBiotech, Birmingham, AL, USA). Images were acquired with an Axio Observer.Z1 inverted microscope (Carl Zeiss, Oberkochen, Germany) with an Axiocam 506 mono camera system. Image processing was performed with Zeiss Zen 3.1 software (blue edition) software.

### Expression of insect cell Flag-HMG20A and Electromobility Shift Assays (EMSAs)

pFASTBAC1 vectors were transformed into DH10Bac bacteria and viruses were generated according to the Bac-to-Bac Baculovirus Expression System protocol (Life Technologies). SF9 cell extracts were prepared 3 days after infection by washing the cells twice with ice cold PBS, resuspending and incubation on ice for 10 min in hypotonic buffer (10 mM HEPES-KOH pH7.9, 1,5 mM MgCl_2_, 10 mM KCl). After 10 sec vortexing and centrifugation for 10 sec each supernatant was aspired. The remaining nuclei were incubated for 20 min in hypertonic buffer (20 mM HEPES-KOH pH 7.9, 25% glycerol 420 mM NaCl, 1,5 mM MgCl_2,_ 0.2 mM EDTA). After centrifugation for 10 min at 16,000 *g*, supernatant was stored at −20 °C or directly used for EMSA. EMSAs were performed as originally described^55^ with the exception that 400 ng of salmon sperm DNA per reaction was used as an unspecific competitor. As EMSA-probe was used, the Cy5 end-labelled double-stranded oligo: CAGGGCTAGTGGATCCCNNNNNNNNNNNNNNTGATTCTGTGGATAACCGTATTACCGCCTTTGAGTGAGCTGATACCGCTCGCGGGCTGCAGGAATTCGA.

### Preparation of nuclear extracts or S1 mononucleosomes, and immunoprecipitation

Preparation was done as previously described^15,16,21^. Briefly, nuclei of 2 × 10^7^ GFP-HMG20A and GFP expressing HK cells were isolated by incubation with 0.3% Triton-X-100 PBS for 10 min at 4 °C and washed three times in PBS, before being resolved in 500 µl freshly prepared Ex100 buffer (10 mM HEPES pH 7.5, 100 mM NaCl, 1.5 MgCl_2_, 10% Glycerol, 10 mM β-Glycerol phosphate, 1 mM DTT, 2 mM Cacl_2_). Chromatin was digested with 150 U micrococcal nuclease (Thermofischer Scientific) for 20 min at 26 °C. The digestion was stopped by adding 10 mM EGTA and transfer to 4 °C. After centrifugation for 10 min at 16,000 *g*, 4 °C supernatants containing mononucleosomes were transferred to fresh reaction tubes. To assess integrity of mononucleosomes 10 µL where are taken, containing DNA fragments isolated using PCR purification columns (QIAGEN) and subjected to agarose gel electrophoresis.

Extracts containing soluble mononucleosomes were incubated with 40 µL GFP-TRAP beads (Chromotek) overnight at 4 °C rotating end over end. Beads were washed twice with 1 ml of 150 mM IP wash buffer 1 (10 mM Tris pH 8.1, 150 mM NaCl, 0.1% Nonidet P40 substitute (v/v)), and 1 ml of 150 mM IP wash buffer 2 ((10 mM Tris pH 8.1, 150 mM NaCl). For immunoblot analysis of precipitated proteins, remaining proteins were eluted by boiling them in 50 µL SDS-loading buffer and compared to input material (5 or 2.5%) by immunoblot. Information on antibodies used is listed in Supplementary Data 4. For label free quantitative mass spectrometry, precipitated proteins were eluted for 30 min at 37 °C, shaking at 1400 rpm in the dark in 50 µl elution buffer (2 M Urea, 50 mM Tris pH 8.1, 2 mM DTT, 20 µg/mL Trypsin (Trypsin Gold, Promega)). Eluted peptides in the supernatant were transferred to a fresh reaction tube. The remaining peptides bound to the beads were alkylated/eluted in 50 µl alkylation buffer (2 M Urea, 50 mM Tris pH 8.1, 2 mM DTT, 20 µg/mL 10 mM chloroacetamide) for 5 min, 37 °C, shaking at 1400 rpm in the dark. Both eluates were combined and eluted peptides were further alkylated and digested by trypsin over night at 25 °C shaking at 800 rpm in the dark. Trypsin digestion was stopped by adding 1% trifluoroacetic acid (Thermo Fischer Scientific). Peptides were subjected to label free quantitative mass spectrometry, comparing peptides originating from GFP against GFP-HMG20A expressing cells. Mass spectrometry experiments were performed twice (biological replicates) with 3 technical replicates each.

### Label-free quantitative Mass-spectrometry (lf-qMS)

Peptides were analyzed by reversed-phase liquid chromatography on an EASY-nLC 1000 or 1200 system (Thermo Fisher Scientific, Odense, Denmark) coupled to a Q Exactive plus or HF mass spectrometer (Thermo Fisher Scientific). HPLC columns of 50 cm length and an inner diameter of 75 µm were in-house packed with ReproSil-Pur 120 C18-AQ 1.9 µm particles (Dr. Maisch GmbH, Germany). Peptide mixtures were separated using linear gradients of 120 or 140 min (total run time + washout) and a two-buffer system: buffer A + + (0.1% formic acid) and buffer B + + (0.1% formic acid in 80% acetonitrile). The mass spectrometer was operated in a data-dependent top 10 or top 15 mode. Peptides were fragmented by higher energy collisional dissociation (HCD) with a normalized collision energy of 27.

### MS Data analysis

The MS raw data were processed using MaxQuant software version 1.4.3.13^56^. Fragmentation spectra were searched against a human sequence database obtained from Uniprot in May 2013 and a file containing frequently observed contaminants such as human keratins. Cysteine carbamidomethylation was set as a fixed modification; N-terminal acetylation and methionine oxidation were set as variable modifications. Trypsin was chosen as specific enzyme, with 2 maximum missed cleavages allowed. The protein and peptide identifications were filtered at 1% FDR. Label-free quantification was performed using the MaxLFQ algorithm^56^ integrated in MaxQuant. The match between runs option was enabled with a matching time window of 0.5 min and an alignment time window of 20 min. All other parameters were left at standard settings. MaxQuant output tables were analysed in Perseus^57^ version 1.5.8.6 as follows: After deleting proteins only identified with modified peptides, hits to the reverse database, contaminants and proteins with one or less razor and unique peptides, label-free intensities were log2 transformed. Proteins were then required to have 3 valid values in at least one triplicate; then the remaining missing values in the data matrix were imputed with values representing a normal distribution around the detection limit of the mass spectrometer. Now, a two-sample t-test was performed to identify proteins enriched in the GFP-HMG20A pull-down compared to the input control. Only those proteins were kept for further analysis. S0 and FDR parameters were set at 0.5 and 0.05, respectively (Supplementary Data 1).

### NuRD binding studies

#### HEK293 cell protein expression

Experiments were carried out as described^27^. Briefly, suspension-adapted HEK Expi293F™ cells (Thermo Fisher Scientific, Waltham, MA, USA, Cat no. #A14527) were cotransfected with combinations of plasmids. Cells were incubated for 65 h at 37 °C with 5% CO_2_ and shaken at 130 rpm, then centrifuged (300 *g*, 5 min) and stored at −80 °C.

### Lysate preparation and immunoprecipitation of GFP-HMG20A and NuRD members

Cell extracts were prepared based on a modified version of a previously described protocol^27^. In brief, cell pellets were lysed in 1 ml of lysis buffer (50 mm Tris/HCl, 500 mm NaCl, 0.1% (v/v) Triton X-100, 1 × cOmplete® EDTA-free protease inhibitor (Roche, Basel, Switzerland), 3 mM MgCl_2_, 1 mM phenylmethylsulfonyl fluoride (PMSF), 3 mM ATP, 10 µg/ml DNase, 10 µg/ml RNase, 1 mm dithiothreitol (DTT), pH 7.9). The lysate was then clarified via centrifugation (20,000 *g*, 20 min, 4 °C); the cleared supernatant was used for GFP-affinity pulldowns as described below.

To prepare GFP beads, streptavidin beads (Thermo Fisher Scientific, Waltham, MA, USA) were first loaded with a GFP nanobody bearing N-terminal 6xHis, SUMO and Streptavidin-Binding Peptide (SBP) tags (the nanobody was expressed and purified from *E. coli* BL21 cells). The GFP-loaded beads were washed using 50 mM HEPES/NaOH, 150 mM NaCl, 0.5% (v/v) IGEPAL® CA630, 1 mM DTT, pH 7.5). GFP nanobody immobilized on beads captured soluble GFP-HMG20A and any partner proteins.

Bound proteins were eluted by 3 × 20 μl treatment with elution buffer (20 mM HEPES/NaOH, 150 mM NaCl, 100 mM biotin, 0.2 mM DTT, pH 8). Eluted fractions were pooled to be analyzed by immunoblotting. Gels were blotted onto PVDF membrane, blocked for 1 h in PBS-T containing 10% (w/v) skim milk and incubated overnight at 4 °C with HRP-conjugated antibodies diluted in PBS-T containing 2% (w/v) skim milk powder. After washes, membranes were imaged using ECL Western Blotting Detection Reagent (GE Healthcare).

### Migration assay

The NCC migration assay was applied according to a published protocol in which *Xenopus* NCC explants were monitored in vitro on petri dishes^58^. Likewise, on differentiation Day9, each EB, considered as an NCC explant, was carefully transferred to gelatine-coated 96-well plates and cultured for at least 24 h. The migration was monitored by manually acquiring a microscopy picture of each attached EB. The migration ability was evaluated by cell velocity and general morphology.

### ChIP-seq

ChIP-seq and ChIP-qPCR was performed as previously described^59^ with the difference that 1 ×10^7^ cells were crosslinked in 2 ml culturing medium with 1% formaldehyde. Information about used qPCR primers are listed in Supplementary Data 4.

Analysis of ChIP-seq data as well as the analysis of chromatin states using public data was performed as previously described^15^.

### Analysis of publicly available ChIP-seq in mESC

Raw sequencing reads were downloaded as FASTQ files from GEO or ENCODE (as described above). These raw FASTQ files were analyzed like the CUT&RUN (trimming, alignment, removal of PCR duplicates). Based on those filtered binary alignment maps (BAMs) normalized coverage tracks (bigWigs) were generated using deepTools bamCoverage function.

Public data sets used in this study:

H3K4me3, PWWP2A, H2A.Z.1 and H2A.Z.2 data from HeLaK cells were used as previously deposited at GEO (“GSE78009”). ChIP-seq data for additional histone modifications was downloaded from the ENCODE portal at UCSC (http://hgdownload.soe.ucsc.edu/goldenPath/hg19/encodeDCC/wgEncodeBroadHistone/). HeLa DNase I hypersensitive sites and mESC histone modification data was downloaded from Encode via the web interface (https://www.encodeproject.org). Data for LSD1, H2A.Z, MTA1 and CHD4 ChIP-seq in mESC was downloaded from GEO. See Supplementary Data 5 for details.

### Plotting and statistics^60,61^

Manipulation of sequencing reads was done using Rsamtools^62^ and genomic intervals were represented as GenomicRanges objects^63^. The analysis of the association between peak intervals and known genomic annotation feature were done using the ChIPseeker package^64^ with default setting using the UCSC hg19 gene definitions (BioConductor package TxDb.Hsapiens.UCSC.hg19.knownGene). As statistical tests, we performed Wilcoxon rank sum tests. The code underlying our analysis is available upon request.

### RT-qPCR and mRNA-seq

RNA isolation, RT-qPCR, and mRNA sequencing was performed as previously described^59^. With the exception of mESC mRNA sequencing, which was performed at Novogene (UK). Information on the qPCR primers used is listed in Supplementary Data 4.

### mRNA-seq analysis

Trimming was performed identical to the CUT&RUN data. Alignment of the trimmed FASTQ files against the mm9 genome (or hg19 for HeLa data) was performed using hisat2 v.2.2.1 with “–min-intronlen 30 –max-intronlen 3000” parameters. The following analysis steps were performed within R v.4.1.2^65^ using a modified version of R/BioConductor package systemPipeR^66^ for various steps. Based on the BAM files and the mouse mm9 GTF (or hg19 GTF for HeLaK data) read counts per gene for each sample were calculated using the summarizedOverlaps function of the GenomicAlignments^63^ R package. The resulting read counts were normalized using DESeq2 v.1.28.1^67^. DESeq2 was used for the identification of differentially expressed genes (log2FC > 2 or log2FC < −2 for mESCs and log2FC > 0.8 or log2FC < −0.8 for HeLaK and adjusted *p*-value < 0.05) for the displayed contrasts, unless otherwise indicated. The PCA was calculated using DESeq2 and plotted using ggplot2^68^. The z-scaled heatmap was clustered according to the Euclidian distance using the “ward.D2” method. Line plots for the gene expression at different days were min-max normalized based on all expression values for each gene. Snapshots based on the coverage tracks were generated using the Gviz package^61^. Gene ontology analysis for the genes of different clusters was performed using Metascape^69^ web interface (www.metascape.org) and plotted using ggplot2.

### Primers

See Supplementary Data 4.

### Xenopus laevis experiments

All procedures involving *Xenopus* embryos were performed according to the German animal use and care law (Tierschutzgesetz) and approved by the German state administration Hesse (Regierungspräsidium Giessen, A 16/201*7)*. All embryos were analyzed before sex determination. *Xenopus laevis* staging, microinjection, lacZ staining, and whole mount in situ hybridization were performed as previously described^70^. For microinjections, capped sense mRNA *(lacZ, mbGFP*^71^ and *mbRFP*^72^*)* and the following Morpholino Oligonucleotides (MO) were used: standard control morpholino (*co MO*, 5′-CCTCTTACCTCAGTTACAATTTATA-3′, Gene Tools, LLC) and *hmg20a* translation blocking MO (*hmg20a* MO, 5′- TGCAGAGGCTGTGCTTTCCATCTAG-3′, Gene Tools, LLC). 10 ng MO were injected into one blastomere of two-cell stage embryos. The spatial expression pattern of *hmg20a* was characterized using albino embryos; sense controls were analyzed for all documented stages. Histological sections were obtained as previously described^73^. For the phenotypical characterization of craniofacial and heart structures, 80 pg *lacZ* RNA, 50 pg *mbGFP* RNA or 80 pg *mbRFP* RNA were coinjected as lineage tracers to mark the injected side. In addition, for rescue experiments human *HMG20A* DNA (130 pg for NC defects, 100 pg for cartilage defects) was co-injected. NC migration was assessed at stage 26-28 by whole mount in situ hybridization, cartilage development at stage 44 by whole-mount immunofluorescence staining^74^. Cartilage phenotypes were quantified by measuring the area of the ceratohyal cartilage using ImageJ’s polygon function. The ratio between the relative surface area of the Morpholino-injected side and the control side was calculated and plotted in a box plot diagram. For phenotypical and immunofluorescence documentation, a Nikon stereo microscope (SMZ18) with a DS-Fi3 Nikon camera and NIS-Elements imaging software was used.

### CUT&RUN

#### Preparation of samples

CUT&RUN was performed on 5×10^5^ mESCs at primed stage of cardiomyocyte differentiation protocol (Fig. 5D) applying the CUTANA® CUT&RUN Kit (version 2) according to the manufacturer’s instructions. Information on antibodies used is listed in Supplementary Data 4. CUT&RUN sequencing libraries were generated using NEBNext® Ultra™ II DNA Library Prep Kit for Illumina® (New England Biolabs) according to the manufacturer’s instruction. Sequencing was performed at Novogene (UK).

### Bioinformatic analysis

Paired end raw FASTQ files were quality and adaptor trimmed using trimGalore v.1.18^75^. Trimmed FASTQ files were aligned against the mouse mm9 reference genome (Illumina’s iGenomes) using hisat2 v.2.2.1^76^ with the “–no-spliced-alignment” parameter and stored as Binary Alignment Map (BAM) files. PCR duplicated reads were removed from BAM files using Picard tools v.2.21.9 (http://picard.sourceforge.net). The resulting BAM files were used to generate individual coverage tracks (bigWig) for each sample using deepTools bamCoverage function^77^. MACS2 v.2.2.7.1^78^ with IGG from wild type or DP as an input was used for the narrow peak calling on the two wild type and two *Hmg20a* DP samples. Only peaks from the wild type samples that were not identified in one of the *Hmg20a* DP samples were used as the real HMG20A binding sites. Additionally, those sites were filtered for known mouse mm9 blacklisted regions^79^. Based on those 2,545 bona fide Hmg20a sites and the individual coverage tracks (bigWigs) for each sample deepTools computeMatrix and plotHeatmap commands were used to generate the binding heatmaps. ChIPseeker^64^ with the UCSC’s mm9 Gene transfer format (GTF) files was used to identify the genomic features or the nearest genes (distance to TSS) that are associated with Hmg20a binding sites. MEME-Suite was used for the motif discovery analysis of the Hmg20a binding sites.

### ATAC-seq

#### Preparation of samples

100,000 primed mESC were harvested and the ATAC-seq kit (Active Motif Cat. 53150) was applied according to the manufacturer’s instruction. Sequencing was performed at Novogene (UK).

### Bioinformatic analysis

Paired end raw ATAC-seq FASTQ files were trimmed, aligned and filtered for PCR duplicates identical to the CUT&RUN data. Peak calling for each BAM file was performed using MACS2 v.2.2.7.1 without input and “-g 2.8e9 -q 0.01 --nomodel” as parameters. Only peaks that were conserved in at least two out of four samples (WT_1, WT_2, PR_1, PR_2) and not overlapping with backlisted regions were counted as real signals. The number of sequencing reads at these ATAC-seq signals were calculated using the summarizedOverlaps function with the “mode = ”Union” parameter (GenomicAlignments package). These raw read counts were normalized and differentially accessible regions (DARs) were calculated using DESeq2. These normalization factors were used to generate normalized coverage tracks (bigWigs) using deepTools bamCoverage function. Heatmaps and average plots were generated identical to CUT&RUN data. The fGSEA package was used to generate the “GSEA” (Supplement Fig. 7C). Here the ATAC-seq signals that were associated with significant deregulated genes (mRNA-seq) using ChIPseeker and used as the “pathways” and the Wald’s t-test (DESeq2) for all ATAC-seq signals were used as the “ranked gene list”.

### Statistics and reproducibility

*Xenopus* experiments: No statistical method was used to predetermine sample size. Embryo batches were separated into equal-size experimental groups and randomly allocated for injection. No data were excluded from the analyses. The investigators were not blinded to allocation during experiments and outcome assessment. Statistical analysis was performed using GraphPad Prism 9.

Human and mouse cell experiments: No statistical method was used to predetermine sample size. No data were excluded from the analyses. The experiments were not randomized.

### Reporting summary

Further information on research design is available in the Nature Portfolio Reporting Summary linked to this article.

## Supplementary information

Supplementary Information

Description of Additional Supplementary Files

Supplementary Data 1

Supplementary Data 2

Supplementary Data 3

Supplementary Data 4

Supplementary Data 5

Supplementary Movie 1

Supplementary Movie 2

Supplementary Movie 3

Supplementary Movie 4

Reporting Summary

## Data Availability

All sequencing data sets (ChIP, mRNA, CUT&RUN and ATAC) specifically collected in this publication have been deposited in NCBI’s Gene Expression Omnibus^80^ and are accessible through GEO Series accession number GSE202199. The mass spectrometry proteomics data have been deposited to the ProteomeXchange Consortium via the PRIDE^81^ partner repository with the dataset identifier PXD038968. Source data are provided with this paper.

## Source data

Source Data

## Code availability

Code used for the analysis is based on publicly available packages (DESeq2, ChIPseeker, etc.) and is well described in these packages and their corresponding vignettes. Scripts to reproduce the data will be made available upon request.
