# Supplementary material for: The H2A.Z and NuRD associated protein HMG20A controls early head and heart developmental transcription programs
Source: Nat Commun. 2023 Jan 28;14:472. doi: 10.1038/s41467-023-36114-x (PMC9884267; doi:10.1038/s41467-023-36114-x)
Supplement: Supplementary file 1 — Supplementary Information [file 41467_2023_36114_MOESM1_ESM.pdf]

Supplementary Information

to

The H2A.Z and NuRD associated protein HMG20A controls early head and heart developmental transcription programs

Andreas Herchenröther[1], Stefanie Gossen[2,+], Tobias Friedrich[3,4,+], Alexander Reim[5], Nadine Daus[1], Felix Diegmüller[1], Jörg Leers[1], Hakimeh Moghaddas Sani[6], Sarah Gerstner[2], Leah Schwarz[2], Inga Stellmacher[1], Laura Victoria Szymkowiak[1,7], Andrea Nist[8], Thorsten Stiewe[8], Tilman Borggrefe[3], Matthias Mann[5], Joel P. Mackay[6], Marek Bartkuhn[4,*], Annette Borchers[2,*], Jie Lan[1,*] and Sandra B. Hake[1,*]

[1]Institute for Genetics, Justus-Liebig University Giessen, 35392 Giessen, Germany
[2]Department of Biology, Molecular Embryology, Philipps University Marburg, 35032 Marburg, Germany
[3]Institute for Biochemistry, Justus-Liebig University Giessen, 35392 Giessen, Germany
[4]Biomedical Informatics and Systems Medicine, Science Unit for Basic and Clinical Medicine, Institute for lung health, Justus-Liebig University Giessen, 35392 Giessen, Germany
[5]Department of Proteomics and Signal Transduction, Max-Planck Institute of Biochemistry, 82152 Martinsried, Germany
[6]School of Life and Environmental Sciences, University of Sydney, New South Wales 2006, Australia
[7]Institute for Physiological Chemistry, Technical University Dresden, 01307 Dresden, Germany
[8]Genomics Core Facility, Institute of Molecular Oncology, Universities of Giessen and Marburg Lung Center, Member of the German Center for Lung Research (DZL), Philipps-University Marburg, 35043 Marburg, Germany

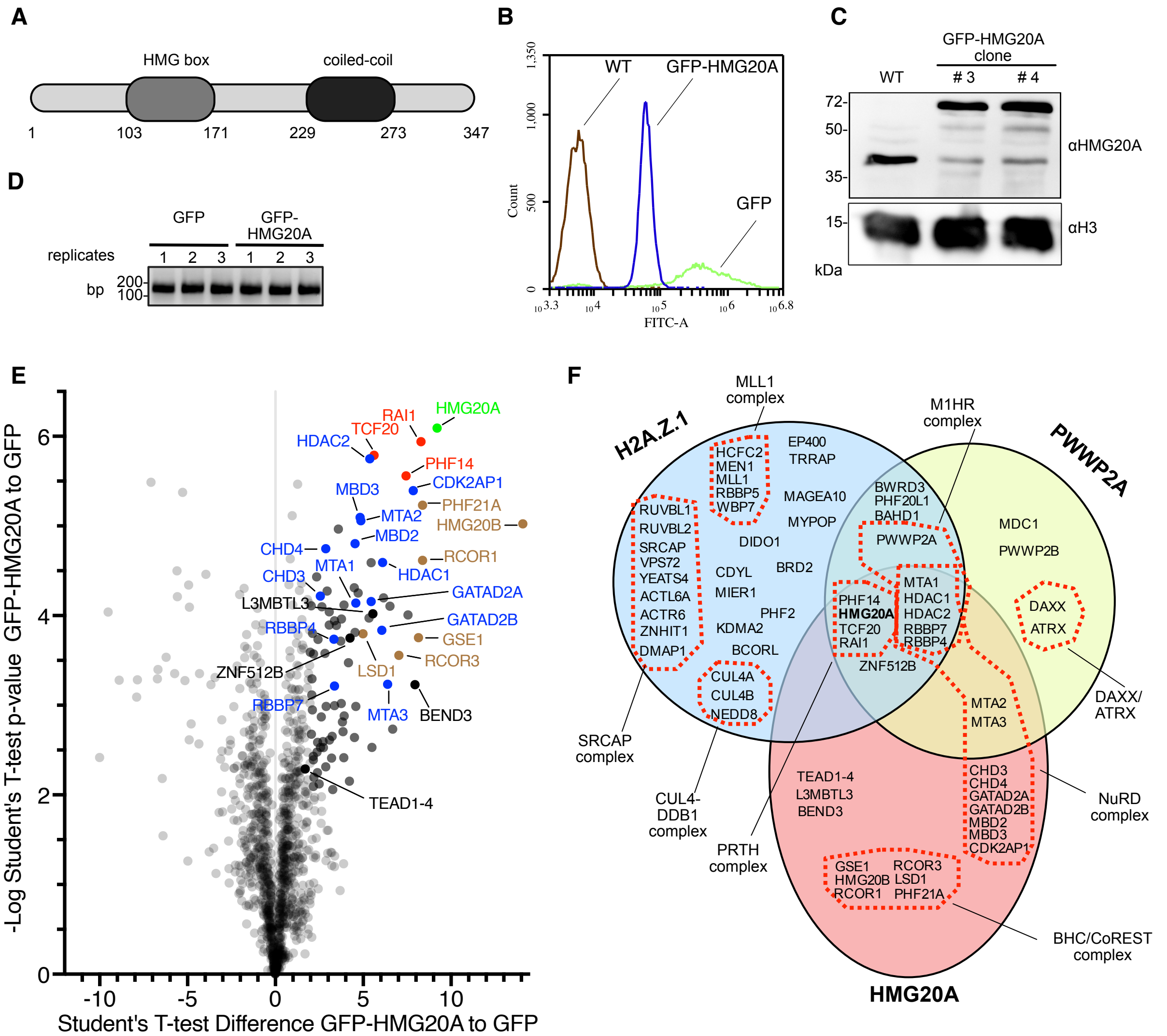

Supplementary Figure 1: HMG20A binds chromatin modifying complexes.

(A) Schematic depiction of human HMG20A protein with its N-terminal HMG box and C-terminal coiled-coil (CC) domain.

(B) Flow cytometry analysis of HeLaK cells (WT, brown) stably expressing GFP (green) or GFP-HMG20A (blue).

(C) Immunoblot of cell extracts from HeLaK cells (WT) stably expressing GFP or GFP-HMG20A with anti-HMG20A antibodies. Anti-H3 serves as loading control.

(D) Agarose gel of purified DNA fragments after MNase digestion using HeLaK cells stably expressing GFP and GFP-HMG20A.

(E) Volcano plot of second replicate of label-free interaction partners of GFP-HMG20A-associated mononucleosomes. Significantly enriched proteins over GFP-associated

mononucleosomes are shown in upper right part. T-test differences were obtained by two-sample t-test. HMG20A is highlighted in bright green, PRTH members in red, BHC/CoREST members in brown, NuRD members in blue, other proteins in black and background binding proteins in grey. See also Figure 1C for Volcano plot of first biological replicate and Supplemental Table 1 for detailed list of HMG20A binders.

(F) Schematic depiction of overlapping H2A.Z.1[1,2], PWWP2A[3] and HMG20A interactomes.

Experiments in C, D were repeated independently three times with consistency. Source data for these figures are provided as a Source Data file.

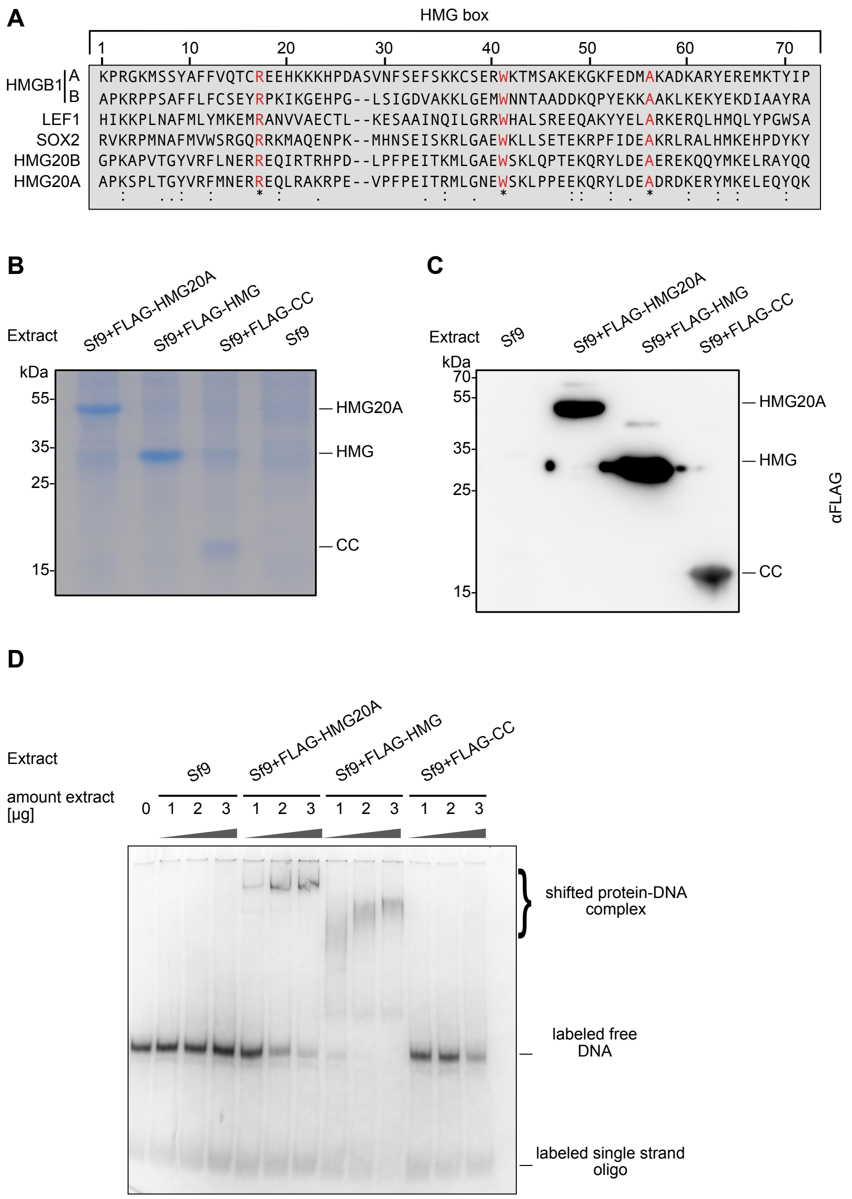

Supplementary Figure 2: Expression and purification of HMG20A deletion proteins.

(A) Alignment of HMG box amino acid sequences from diverse human HMG-box containing proteins. Alignment was performed using Clustal Omega.

(B, C) Coomassie-stained SDS-PAGE gel (**B**) or anti-FLAG immunoblot **(C)** of extracts from Sf9 cells expressing FLAG-tagged HMG20A, HMG or CC proteins. Shown is one representative gel/blot of two consistent experiments.

(D) Electrophoretic mobility shift assay (EMSA) of a Cy5-labeled DNA probe together with whole Sf9 extracts containing different FLAG-HMG20A proteins (see above) in

increasing amounts. EMSA with whole cell extracts of uninfected Sf9 cells served as negative control.

Source data for these figures are provided as a Source Data file.

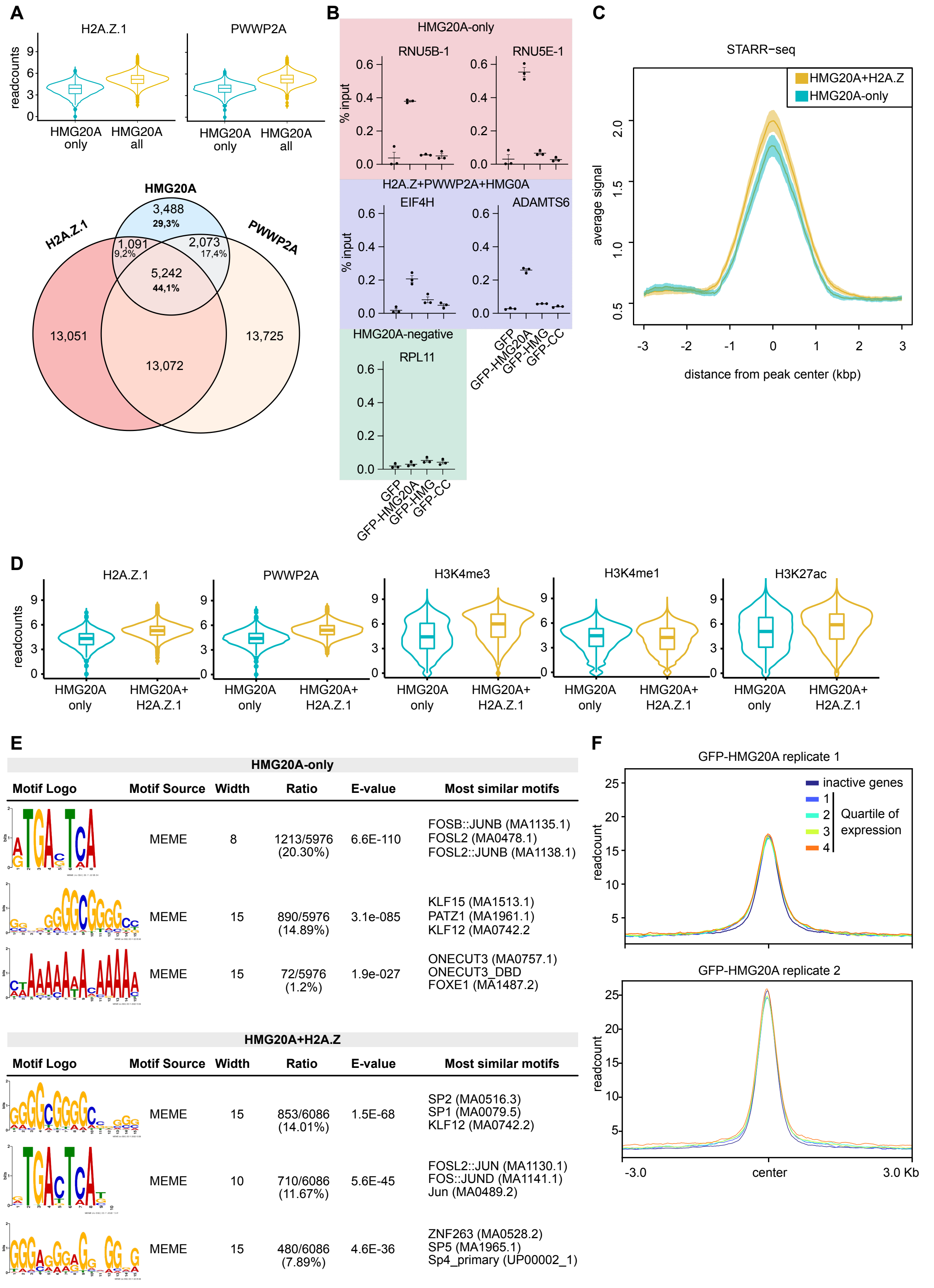

Supplementary Figure 3: HMG20A binds regulatory genomic regions but is not correlated to transcriptional output in HeLaK cells

(A) Top: Violin plots of GFP-H2A.Z.1 (left) and GFP-PWWP2A (right) ChIP-seq read counts at HMG20A-only (blue) or HMG20A+H2A.Z (yellow) binding sites. Bottom: Venn diagram displaying numbers of GFP-H2A.Z.1, -PWWP2A and -HMG20A ChIP-seq binding sites and their overlap.
(B) Validation of ChIP-seq data by ChIP-qPCR at selected loci. Shown is percent input of three biological replicates of GFP, GFP-HMG20A, -HMG or -CC ChIP-qPCR of HMG20A-only sites (red: RNUB-1 and RNUE-1downstream; see red bar in Figure 3A as example), HMG20A+H2A.Z.1-positive sites (purple: EIF4H promoter and ADAMTS3 gene body; see blue bar in Figure 3A as example) and an HMG20A/H2A.Z.1-negative site (green: RPL11 gene body; see green bar in Figure 3A as example) as negative control. Data is presented as mean ± SEM of three biological replicates.
(C) Enrichment plot depicting accumulation of published STARR-seq signals[4] - which correspond to enhancers - at HMG20A-only (blue) or HMG20A+H2A.Z (yellow) ChIP-seq regions.
(D) Violin plots of GFP-H2A.Z.1, GFP-PWWP2A, H3K4me3 (promoter), H3K4me1 (enhancer), H3K27ac (active regulatory regions) ChIP-seq read counts at HMG20A+H2A.Z (yellow) or HMG20A-only (blue) binding sites.
(E) Top-enriched motifs within HMG20A-only (top) or within HMG20A+H2A.Z (bottom) ChIP-seq peaks identified with MEME-ChIP**.**
(F) Average binding plots of both replicates of ChIP-seq identified GFP-HMG20A binding sites. The line colors reflect the average binned expression levels of associated genes.
Source data for these figures are provided as a Source Data file.

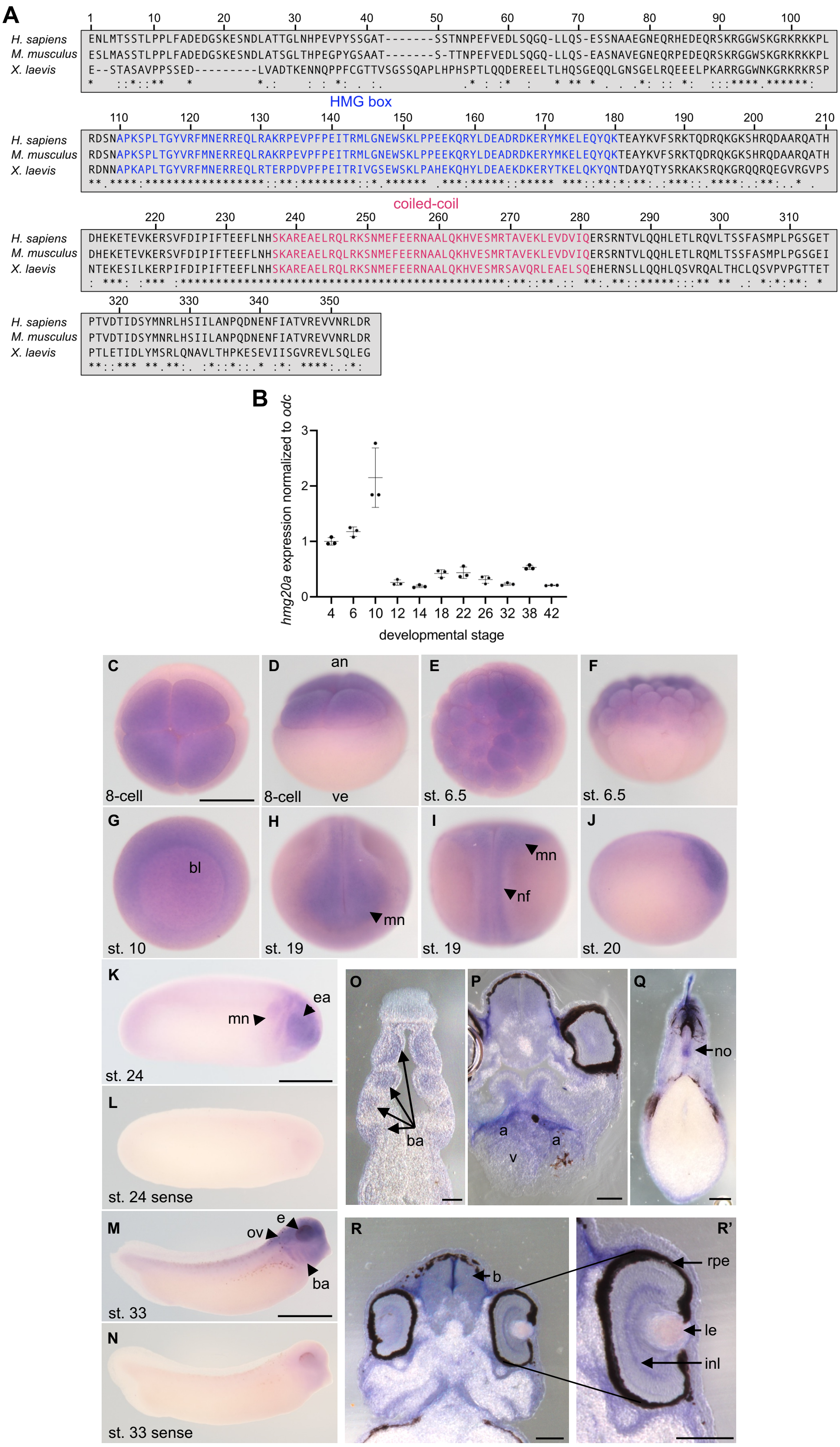

Supplementary Figure 4: *hmg20a* expression in *X. laevis*.

(A) Alignment of human (*H. sapiens*), mouse (*M. musculus*) and frog (*X. laevis*) Hmg20a protein sequences. HMG box is shown in blue, coiled-coil region in red. Alignment was performed using Clustal Omega.

(B) Temporal expression pattern of *Xenopus hmg20a:* RT-qPCR of *hmg20a* mRNA expression covering *X. laevis* developmental stages 4 (8-cell stage) to 42 normalized to *odc* expression. Error bars indicate s.e.m. of three technical replicates.

(C-R') Spatial expression pattern of *hmg20A* determined by whole mount *in situ* hybridization. *hmg20A* mRNA is detected at early stages of *Xenopus laevis* development. **(C)** 8-cell stage embryo, anterior view. **(D)** 8-cell stage embryo, dorso-lateral view, animal and vegetal pole are indicated. **(E)** Embryo at blastula stage 6.5. anterior view. **(F)** Same embryo as in **E**, dorsal view. **(G)** Embryo at gastrula stage 10. **(H)** Embryo at neurula stage 19, anterior view. **(I)** Same embryo as in **H**, dorsal view. **(J)** Embryo at stage 20, lateral view. **(K)** Embryo at stage 24, lateral view. **(L)** Sense control, embryo at stage 24. **(M)** Embryo at stage 33, lateral view. **(N)** Sense control, embryo at stage 33. Scale bar in **C-N** is 1mm. **(O)** Transverse section through the branchial arch region of a stage 31 embryo, *hmg20A* expression in the branchial arches is indicated by arrows. **(P-R')** Transverse sections of a stage 42 embryo. **(P)** *hmg20A* is partially expressed in the heart region. **(Q)** *hmg20A* expression within the notochord (no). **(R, R')** *hmg20A* is partially expressed in the brain and eye. Scale bar in **O-R'** is 100 µm. abbreviations: a, atrium, an, animal; b, brain; ba, branchial arches; bl, blastoporus; ea, eye anlage; e, eye; inl, inner nuclear layer; le, lens; mn, migratory neural crest; nf, neural fold; no, notochord; ov, otic vesicle; rpe, retinal pigment epithelium v, ventricle; ve, vegetal.

Source data for these figures are provided as a Source Data file.

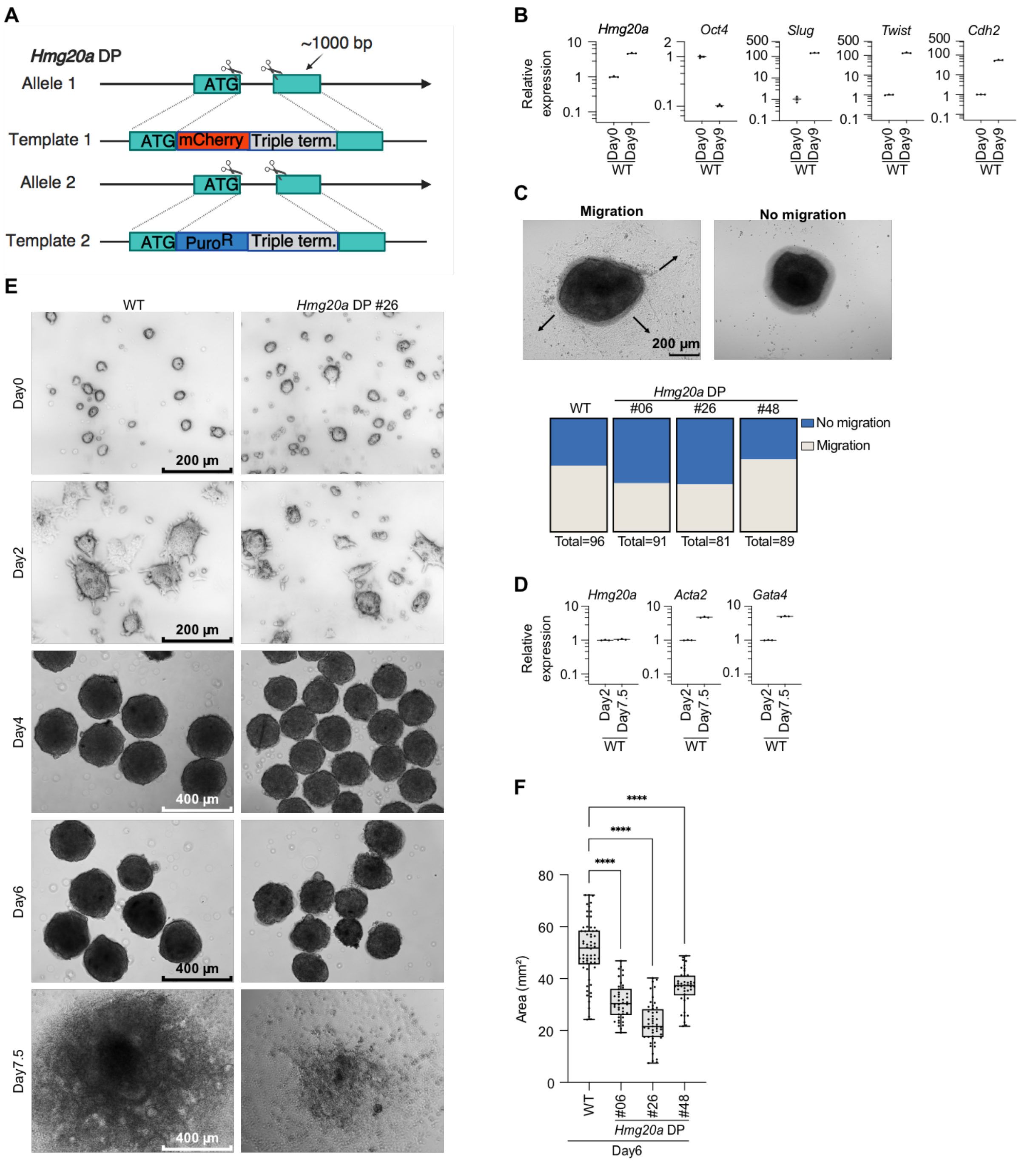

Supplementary Figure 5: Loss of HMG20A impairs mESC differentiation

(A) Schematic depiction of *Hmg20A* DP generation in mESCs by introducing mCherry_triple terminator sites and a puromycin resistance_triple terminator site into both *Hmg20a* alleles directly after the start codon by a CRISPR/Cas9-based approach. Created with BioRender.com.

(B) RT-qPCR of EMT marker genes *Slug, Twist Cdh2,* pluripotency marker *Oct4* and *Hmg20a* in WT cells at Day0 and Day9 of neural crest differentiation protocol.

Expression was normalized to *Hprt, 18S RNA and Gapdh* expression. Data is presented as mean ± SEM of three technical replicates.

(C) Migration assay. Top: Representative microscopy pictures of EBs at Day9 of neural crest differentiation protocol depicting migrating (left, see arrows) or not migrating (right) cells. Bottom: Quantification of migration capability of cells from WT and three *Hmg20a* DP embryoid body (EB) clones based on visual inspection (see top pictures). (Chi-square, two-sided p= 0.0344 (#06), p = 0.0301 (#26), p = 0.4260 (#48)).

(D) RT-qPCR of cardiomyocyte marker genes *Acta2* (middle) *and Gata4* (right) in WT cells at Day2 and Day7.5 of neural crest differentiation protocol. Expression was normalized to *Hprt, 18S RNA and Gapdh* expression. Data is presented as mean ± SEM of three technical replicates.

(E) Phase-contrast microscopy images of WT and *Hmg20a* DP clone #26 mESCs and EBs during cardiomyocyte differentiation.

(F) Size of EBs of WT and three individual *Hmg20a* DP cells at Day6 of cardiomyocyte differentiation protocol. Number of measured EBs indicated above. (Man-Whitney, two-tailed p= 3.6101185369e-015 (#06), p = 5.4103237427e-023 (#26), p = 4.2379561787e-010 (#48)). Error bars indicate min/max values, lines indicate median, boxes indicate 25th to 75th percentiles. Data was derived from two independent differentiation approaches.

Experiments in E, F were repeated independently two times with consistency.
Source data for these figures are provided as a Source Data file.

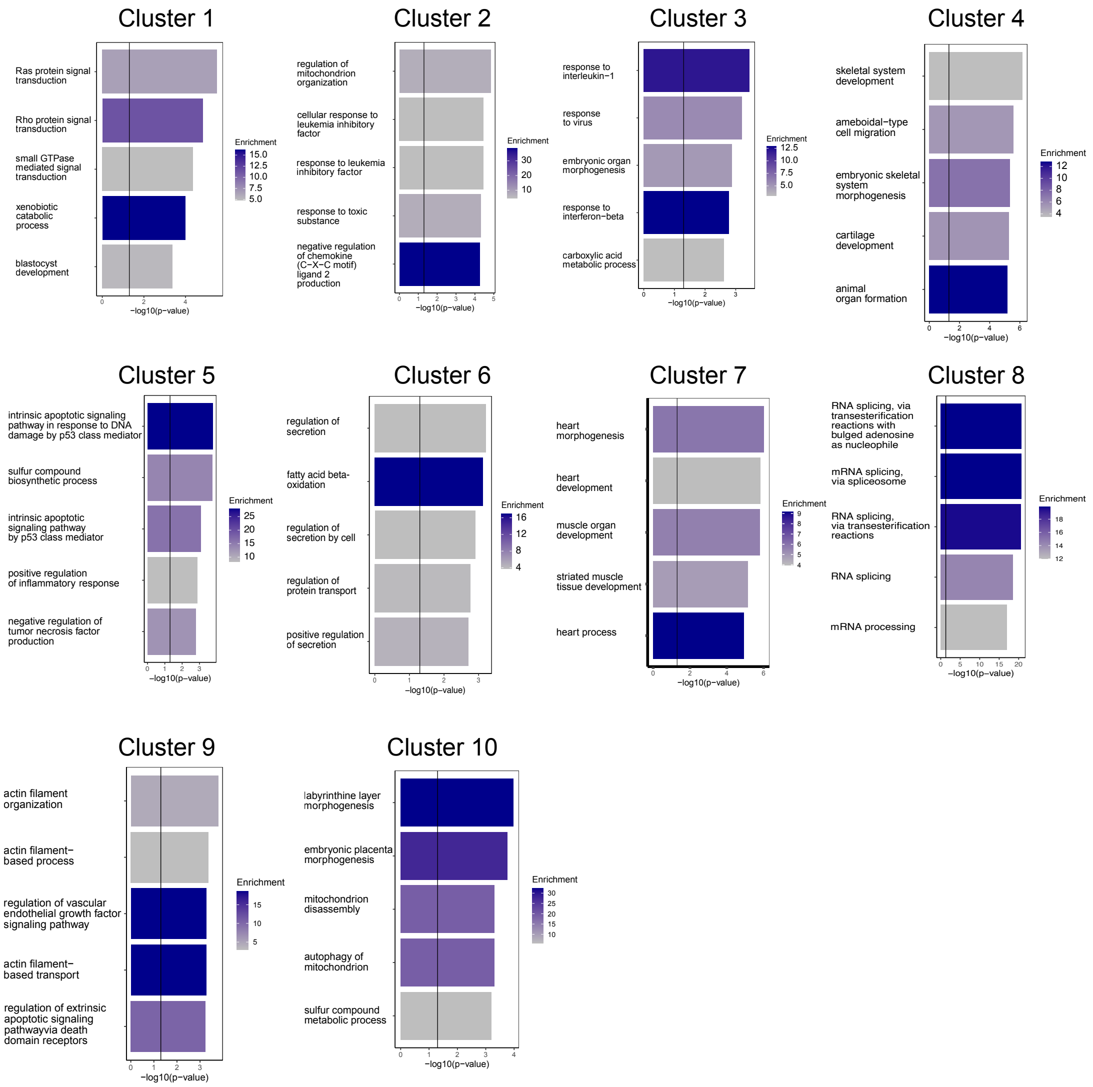

Supplementary Figure 6: HMG20A regulates early transcription programs of cardiomyocyte differentiation.

GO term analysis of deregulated genes upon HMG20A depletion within 10 clusters identified in Figure 6C.

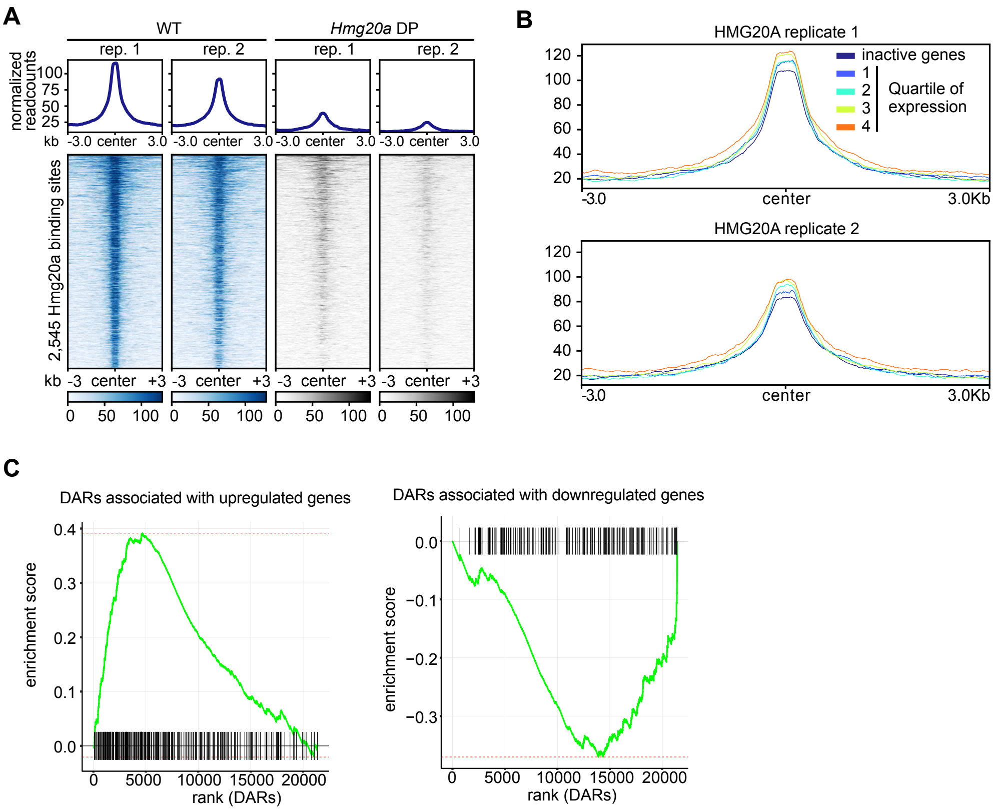

Supplementary Figure 7: HMG20A is associated with transcriptionally active genes.

(A) Density heatmap of 2,545 HMG20A binding sites detected in CUT&RUN (see Figure 7A). Color intensity represents normalized and globally scaled tag counts.

(B) Average binding plots of both replicates of CUT&RUN identified HMG20A binding sites (see Supplemental Figure 7A). The line colors reflect the average binned expression levels of associated genes.

(C) Gene set enrichment plot of genes associated with differentially accessible regions (DARs) after HMG20A depletion correlated to gene expression. Notice that more open accessibly sites correlate with increase in gene transcription (left; padj = 5.502329e-16; NES = 2.2991133), while more inaccessible sites correlate with reduction in gene expression (right; padj = 1.620638e-04; NES = -1.6649125)[5].

HMG20A-only

Motif Logo	Motif Source	Width	Ratio	E-value	Most similar motifs
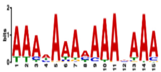	MEME	15	214/1677<br>(12.76%)	1.1E-164	ZNF384 (MA1125.1)<br>Mtf1_secondary (UP00097_2)<br>Zfp105_primary (UP00037_1)
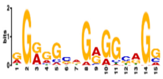	MEME	15	258/1677<br>(15.38%)	4.7e-070	ZNF263 (MA0528.1)<br>Zfp281_primary (UP00021_1)<br>SP2 (MA0516.1)
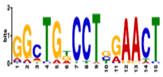	MEME	15	78/1677<br>(4.65%)	2.7e-053	Nr5a2 (MA0505.1)<br>ZNF306_full

HMG20A+H2A.Z

Motif Logo	Motif Source	Width	Ratio	E-value	Most similar motifs
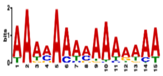	MEME	15	66/868<br>(7.60%%)	2.7E-92	ZNF384 (MA1125.1)<br>Srf_secondary (UP00077_2)<br>Zfp105_primary
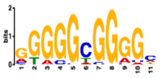	MEME	11	162/868<br>(18.66%)	7.1E-33	SP1 (MA0079.3)<br>SP2 (MA0516.1)<br>SP1_DBD
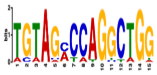	MEME	15	17/868<br>(1.95%)	3.1E-15	

Supplementary Figure 8: HMG20A localizes to specific DNA-sequences in mESCs

Top-enriched motifs within HMG20A-only (top) or within HMG20A+H2A.Z (bottom) CUT&RUN peaks identified with MEME-ChIP.

Supplementary References

1 Punzeler, S. *et al.* Multivalent binding of PWWP2A to H2A.Z regulates mitosis and neural crest differentiation. *EMBO J* (2017).

2 Vardabasso, C. *et al.* Histone Variant H2A.Z.2 Mediates Proliferation and Drug Sensitivity of Malignant Melanoma. *Molecular cell* **59**, 75-88 (2015).

3 Link, S. *et al.* PWWP2A binds distinct chromatin moieties and interacts with an MTA1-specific core NuRD complex. *Nat Commun* **9**, 4300 (2018).

4 Muerdter, F. *et al.* Resolving systematic errors in widely used enhancer activity assays in human cells. *Nat Methods* **15**, 141-149 (2018).

5 Korotkevich, G. *et al.* Fast gene set enrichement analysis. *bioRxiv* **doi:** https://doi.org/10.1101/060012 (2021).
