# Supplementary material for: The H2A.Z and NuRD associated protein HMG20A controls early head and heart developmental transcription programs
Source: Nat Commun. 2023 Jan 28;14:472. doi: 10.1038/s41467-023-36114-x (PMC9884267; doi:10.1038/s41467-023-36114-x)
Supplement: Supplementary file 2 — Description of Additional Supplementary Files [file 41467_2023_36114_MOESM2_ESM.pdf]

Description of Additional Supplementary Files

File Name: Supplementary Data 1
Description: Excel list of proteins associated with GFP-HMG20A identified by label-free quantitative mass spectrometry.

File Name: Supplementary Data 2
Description: mRNA-seq data showing differentially expressed genes in HeLaK cells transfected with HMG20A siRNA pool compared to control siRNA pool.

File Name: Supplementary Data 3
Description: mRNA-seq data sowing differentially expressed genes in *Hmg20a* DP mESCs compared to control mESCs at distinct days during CM differentiation.

File Name: Supplementary Data 4
Description: Lists of used antibodies, primers and oligos for guide RNAs.

File Name: Supplementary Data 5
Description: List of used public ChIP-seq data files.

File Name: Supplementary Movies 1-4
Description: Life-cell imaging of WT (**A**) or *Hmg20a* DP clones #06 (**B**), #26 (**C**) and #48 (**D**) mESCs at CM differentiation protocol Day7.5. 20x magnification.
