# Supplementary material for: The H2A.Z and NuRD associated protein HMG20A controls early head and heart developmental transcription programs
Source: Nat Commun. 2023 Jan 28;14:472. doi: 10.1038/s41467-023-36114-x (PMC9884267; doi:10.1038/s41467-023-36114-x)
Supplement: Supplementary file 7 — Supplementary Data 5 [file 41467_2023_36114_MOESM7_ESM.pdf]

Supplementary Data 5

Public ChIP-seq data	downloaded files
HeLa H3K4me1	“wgEncodeBroadHistoneHelas3H3k04me1StdRawDataRep1.fastq.gz[hgdownload.soe.ucsc.edu/goldenPath/hg19/encodeDCC/wgEncodeBroadHistone/wgEncodeBroadHistoneHelas3H3k04me1StdRawDataRep1.fastq.gz]”
HeLa H3K4me3	“wgEncodeBroadHistoneHelas3H3k4me3StdRawDataRep1.fastq.gz[hgdownload.soe.ucsc.edu/goldenPath/hg19/encodeDCC/wgEncodeBroadHistone/wgEncodeBroadHistoneHelas3H3k4me3StdRawDataRep1.fastq.gz]”
HeLa H3K27ac	“wgEncodeBroadHistoneHelas3H3k27acStdAlnRep1.fastq.gz[hgdownload.soe.ucsc.edu/goldenPath/hg19/encodeDCC/wgEncodeBroadHistone/wgEncodeBroadHistoneHelas3H3k27acStdAlnRep1.fastq.gz]”
HeLa H3K27me3	“wgEncodeBroadHistoneHelas3H3k27me3StdRawDataRep1.fastq.gz[hgdownload.soe.ucsc.edu/goldenPath/hg19/encodeDCC/wgEncodeBroadHistone/wgEncodeBroadHistoneHelas3H3k27me3StdRawDataRep1.fastq.gz]”
HeLa H3K36me3	“wgEncodeBroadHistoneHelas3H3k36me3StdRawDataRep1.fastq.gz[hgdownload.soe.ucsc.edu/goldenPath/hg19/encodeDCC/wgEncodeBroadHistone/wgEncodeBroadHistoneHelas3H3k36me3StdRawDataRep1.fastq.gz]”
HeLa DNaseI-seq	“ENCFF526VFR[https://www.encodeproject.org/files/ENCFF526VFR/]” (Encode)
mESC H3K4me3	“ENCFF001KER[https://www.encodeproject.org/files/ENCFF001KER/]” (Encode)
mESC H3K4me1	“ENCFF001KEF[https://www.encodeproject.org/files/ENCFF001KEF/]” (Encode)
mESC LSD1/KDM1A	“SRR122471[https://www.ncbi.nlm.nih.gov/geo/query/acc.cgi?acc=GSM687283]” (GSM687283)
mESC H2A.Z	“SRR390385[https://www.ncbi.nlm.nih.gov/geo/query/acc.cgi?acc=GSM849928]” (GSM849928)
mESC MTA1	“SRR8236016[https://www.ncbi.nlm.nih.gov/geo/query/acc.cgi?acc=GSM3486610]” (GSM3486610)
mESC CHD4	“SRR1569084[https://www.ncbi.nlm.nih.gov/geo/query/acc.cgi?acc=GSM1499118]” (GSM1499118)
