# Supplementary material for: The H2A.Z and NuRD associated protein HMG20A controls early head and heart developmental transcription programs
Source: Nat Commun. 2023 Jan 28;14:472. doi: 10.1038/s41467-023-36114-x (PMC9884267; doi:10.1038/s41467-023-36114-x)
Supplement: Supplementary file 12 — Reporting Summary [file 41467_2023_36114_MOESM12_ESM.pdf]

nature portfolio

Corresponding author(s): Marek Bartkuhn
Annette Borchers
Jie Lan
Sandra B. Hake

Last updated by author(s): Dec 16, 2022

# Reporting Summary

Nature Portfolio wishes to improve the reproducibility of the work that we publish. This form provides structure for consistency and transparency in reporting. For further information on Nature Portfolio policies, see our Editorial Policies and the Editorial Policy Checklist.

## Statistics

For all statistical analyses, confirm that the following items are present in the figure legend, table legend, main text, or Methods section.

| n/a | Confirmed | |
|---|---|---|
| ☐ | ☒ | The exact sample size (*n*) for each experimental group/condition, given as a discrete number and unit of measurement |
| ☐ | ☒ | A statement on whether measurements were taken from distinct samples or whether the same sample was measured repeatedly |
| ☐ | ☒ | The statistical test(s) used AND whether they are one- or two-sided<br>*Only common tests should be described solely by name; describe more complex techniques in the Methods section.* |
| ☐ | ☒ | A description of all covariates tested |
| ☐ | ☒ | A description of any assumptions or corrections, such as tests of normality and adjustment for multiple comparisons |
| ☐ | ☒ | A full description of the statistical parameters including central tendency (e.g. means) or other basic estimates (e.g. regression coefficient) AND variation (e.g. standard deviation) or associated estimates of uncertainty (e.g. confidence intervals) |
| ☐ | ☒ | For null hypothesis testing, the test statistic (e.g. *F*, *t*, *r*) with confidence intervals, effect sizes, degrees of freedom and *P* value noted<br>*Give P values as exact values whenever suitable.* |
| ☒ | ☐ | For Bayesian analysis, information on the choice of priors and Markov chain Monte Carlo settings |
| ☒ | ☐ | For hierarchical and complex designs, identification of the appropriate level for tests and full reporting of outcomes |
| ☐ | ☐ | Estimates of effect sizes (e.g. Cohen's *d*, Pearson's *r*), indicating how they were calculated |

*Our web collection on statistics for biologists contains articles on many of the points above.*

## Software and code

Policy information about availability of computer code

**Data collection**

Xenopus: NIS-Elements to acquire images of Xenopus embryos
Human and mouse cell lines: qPCR: CFX Maestro Software 2.3
The vast majority of code used for the analysis is based on publicly available packages (DESeq2, ChIPseeker, etc.) and is well described in these packages and their corresponding vignettes. We have referenced these packages in the M&M sections. The final figures were generated using either the well-described ggplot2 package or R base functions. Our analyses are with-in the scope of typical NGS-based expression or epigenomics studies, including differential expression or chromatin binding analyses.
We clarify that our code does not contain novel techniques, algorithms, or complex analysis workflows, but only contains basic code that is needed for the work within R.

**Data analysis**

Xenopus: ImageJ and GraphPad Prism for Xenopus data analysis

Human and mouse cell lines: Immunofluorescence microscopy: Zeiss Zen 3.1 software; qPCR: Microsoft Excel 2016 (Data Analysis) and GraphPad Prism 9 (Data analysis/Data Visualisation)

FastQC (https://www.bioinformatics.babraham.ac.uk/projects/fastqc/)
Trim Galore (https://www.bioinformatics.babraham.ac.uk/projects/trim_galore/)
BWT index from Illumina's iGenome repository (https://emea.support.illumina.com/sequencing/, sequencing_software/igenome.html)
bowtie version 1.1.2

hisat2 v.2.2.171

```
Integrative Genome Viewer (IGV),
MACS2 2.2.7.1
R version 4.1.2 (2021-11-01)
Platform: x86_64-apple-darwin17.0 (64-bit)
Running under: macOS Catalina 10.15.7

Matrix products: default
BLAS:   /System/Library/Frameworks/Accelerate.framework/Versions/A/Frameworks/vecLib.framework/Versions/A/libBLAS.dylib
LAPACK: /Library/Frameworks/R.framework/Versions/4.1/Resources/lib/libRlapack.dylib

Random number generation:
 RNG:     Mersenne-Twister
 Normal:  Inversion
 Sample:  Rounding

locale:
[1] en_US.UTF-8/en_US.UTF-8/en_US.UTF-8/C/en_US.UTF-8/en_US.UTF-8

attached base packages:
[1] grid      stats4    stats     graphics  grDevices utils     datasets  methods   base

other attached packages:
 [1] clusterProfiler_4.2.2       GeneOverlap_1.30.0          data.table_1.14.2           Hmisc_4.6-0
 [5] ggplot2_3.3.5               Formula_1.2-4               survival_3.2-13             lattice_0.20-45
 [9] fields_13.3                 viridis_0.6.2               viridisLite_0.4.0           spam_2.8-0
[13] DESeq2_1.34.0               limma_3.50.0                fts_0.9.9.2                 zoo_1.8-9
[17] rtracklayer_1.54.0          gplots_3.1.1                RColorBrewer_1.1-2          Gviz_1.38.3
[21] ShortRead_1.52.0            GenomicAlignments_1.30.0    SummarizedExperiment_1.24.0 MatrixGenerics_1.6.0
[25] matrixStats_0.61.0          Rsamtools_2.10.0            Biostrings_2.62.0           XVector_0.34.0
[29] BiocParallel_1.28.3         GenomicFeatures_1.46.4      AnnotationDbi_1.56.2        Biobase_2.54.0
[33] GenomicRanges_1.46.1        GenomeInfoDb_1.30.1         IRanges_2.28.0              S4Vectors_0.32.3
[37] BiocGenerics_0.40.0

loaded via a namespace (and not attached):
  [1] utf8_1.2.2             tidyselect_1.1.1       RSQLite_2.2.9          htmlwidgets_1.5.4      scatterpie_0.1.7
  [6] munsell_0.5.0          withr_2.4.3            colorspace_2.0-2       GOSemSim_2.20.0        filelock_1.0.2
 [11] knitr_1.37             rstudioapi_0.13        DOSE_3.20.1            GenomeInfoDbData_1.2.7 hwriter_1.3.2
 [16] polyclip_1.10-0        bit64_4.0.5            farver_2.1.0           downloader_0.4         vctrs_0.3.8
 [21] treeio_1.18.1          generics_0.1.2         xfun_0.29              biovizBase_1.42.0      BiocFileCache_2.2.1
 [26] R6_2.5.1               graphlayouts_0.8.0     locfit_1.5-9.4         AnnotationFilter_1.18.0 bitops_1.0-7
 [31] cachem_1.0.6           fgsea_1.20.0           gridGraphics_0.5-1     DelayedArray_0.20.0    assertthat_0.2.1
 [36] BiocIO_1.4.0           scales_1.1.1           ggraph_2.0.5           nnet_7.3-17            enrichplot_1.14.1
 [41] gtable_0.3.0           tidygraph_1.2.0        ensembldb_2.18.3       rlang_1.0.1            genefilter_1.76.0
 [46] splines_4.1.2          lazyeval_0.2.2         GEOquery_2.62.2        dichromat_2.0-0        checkmate_2.0.0
 [51] yaml_2.2.2             reshape2_1.4.4         backports_1.4.1        qvalue_2.26.0          tools_4.1.2
 [56] ggplotify_0.1.0        ellipsis_0.3.2         Rcpp_1.0.8             plyr_1.8.6             base64enc_0.1-3
 [61] progress_1.2.2         zlibbioc_1.40.0        purrr_0.3.4            RCurl_1.98-1.5         prettyunits_1.1.1
 [66] rpart_4.1.16           ggrepel_0.9.1          cluster_2.1.2          magrittr_2.0.2         DO.db_2.9
 [71] ProtGenerics_1.26.0    hms_1.1.1              patchwork_1.1.1        xtable_1.8-4           XML_3.99-0.8
 [76] jpeg_0.1-9             gridExtra_2.3          compiler_4.1.2         biomaRt_2.50.3         tibble_3.1.6
 [81] maps_3.4.0             shadowtext_0.1.1       KernSmooth_2.23-20     crayon_1.4.2           htmltools_0.5.2
 [86] ggfun_0.0.5            tzdb_0.2.0             tidyr_1.2.0            geneplotter_1.72.0     aplot_0.1.2
 [91] DBI_1.1.2              tweenr_1.0.2           dbplyr_2.1.1           MASS_7.3-55            rappdirs_0.3.3
 [96] Matrix_1.4-0           readr_2.1.2            cli_3.1.1              parallel_4.1.2         dotCall64_1.0-1
[101] igraph_1.2.11          pkgconfig_2.0.3        foreign_0.8-82         xml2_1.3.3             ggtree_3.2.1
[106] annotate_1.72.0        yulab.utils_0.0.4      stringr_1.4.0          VariantAnnotation_1.40.0 digest_0.6.29
```

For manuscripts utilizing custom algorithms or software that are central to the research but not yet described in published literature, software must be made available to editors and reviewers. We strongly encourage code deposition in a community repository (e.g. GitHub). See the Nature Portfolio guidelines for submitting code & software for further information.

## Data

Policy information about availability of data

All manuscripts must include a data availability statement. This statement should provide the following information, where applicable:

- Accession codes, unique identifiers, or web links for publicly available datasets
- A description of any restrictions on data availability
- For clinical datasets or third party data, please ensure that the statement adheres to our policy

All sequencing data sets (ChIP-seq, RNA-seq, CUT&RUN-seq and ATAC-seq) discussed in this publication have been deposited in NCBI's Gene Expression Omnibus80 and are accessible through GEO Series accession number "GSE202199[https://www.ncbi.nlm.nih.gov/geo/query/acc.cgi?acc=GSE202199]".

Public data sets used in this study:
H3K4me3, PWWP2A, H2A.Z.1 and H2A.Z.2 data from HeLaK cells were used as previously deposited at GEO ("GSE78009[https://www.ncbi.nlm.nih.gov/geo/query/acc.cgi?acc=GSE78009]"). ChIP-seq data for additional histone modifications was downloaded from the ENCODE portal at UCSC (http://hgdownload.soe.ucsc.edu/

goldenPath/hg19/encodeDCC/wgEncodeBroadHistone/). HeLa DNase I hypersensitive sites and mESC histone modification data was downloaded from Encode via the web interface (https://www.encodeproject.org). Data for LSD1, H2A.Z, MTA1 and CHD4 ChIP-seq in mESC was downloaded from GEO.

Public ChIP-seq data downloaded files
HeLa H3K4me1 “wgEncodeBroadHistoneHelas3H3k04me1StdRawDataRep1.fastq.gz[hgdownload.soe.ucsc.edu/goldenPath/hg19/encodeDCC/wgEncodeBroadHistone/wgEncodeBroadHistoneHelas3H3k04me1StdRawDataRep1.fastq.gz]”
HeLa H3K4me3 “wgEncodeBroadHistoneHelas3H3k4me3StdRawDataRep1.fastq.gz[hgdownload.soe.ucsc.edu/goldenPath/hg19/encodeDCC/wgEncodeBroadHistone/wgEncodeBroadHistoneHelas3H3k4me3StdRawDataRep1.fastq.gz]”
HeLa H3K27ac “wgEncodeBroadHistoneHelas3H3k27acStdAlnRep1.fastq.gz[hgdownload.soe.ucsc.edu/goldenPath/hg19/encodeDCC/wgEncodeBroadHistone/wgEncodeBroadHistoneHelas3H3k27acStdAlnRep1.fastq.gz]”
HeLa H3K27me3 “wgEncodeBroadHistoneHelas3H3k27me3StdRawDataRep1.fastq.gz[hgdownload.soe.ucsc.edu/goldenPath/hg19/encodeDCC/wgEncodeBroadHistone/wgEncodeBroadHistoneHelas3H3k27me3StdRawDataRep1.fastq.gz]”
HeLa H3K36me3 “wgEncodeBroadHistoneHelas3H3k36me3StdRawDataRep1.fastq.gz[hgdownload.soe.ucsc.edu/goldenPath/hg19/encodeDCC/wgEncodeBroadHistone/wgEncodeBroadHistoneHelas3H3k36me3StdRawDataRep1.fastq.gz]”
HeLa DNaseI-seq “ENCFF526VFR[https://www.encodeproject.org/files/ENCFF526VFR/]” (Encode)
mESC H3K4me3 “ENCFF001KER[https://www.encodeproject.org/files/ENCFF001KER/]” (Encode)
mESC H3K4me1 “ENCFF001KEF[https://www.encodeproject.org/files/ENCFF001KEF/]” (Encode)
mESC LSD1/KDM1A “SRR122471[https://www.ncbi.nlm.nih.gov/geo/query/acc.cgi?acc=GSM687283]” (GSM687283)
mESC H2A.Z “SRR390385[https://www.ncbi.nlm.nih.gov/geo/query/acc.cgi?acc=GSM849928]” (GSM849928)
mESC MTA1 “SRR8236016[https://www.ncbi.nlm.nih.gov/geo/query/acc.cgi?acc=GSM3486610]” (GSM3486610)
mESC CHD4 “SRR1569084[https://www.ncbi.nlm.nih.gov/geo/query/acc.cgi?acc=GSM1499118]” (GSM1499118)

## Human research participants

Policy information about studies involving human research participants and Sex and Gender in Research.

| | |
|---|---|
| Reporting on sex and gender | *Use the terms sex (biological attribute) and gender (shaped by social and cultural circumstances) carefully in order to avoid confusing both terms. Indicate if findings apply to only one sex or gender; describe whether sex and gender were considered in study design whether sex and/or gender was determined based on self-reporting or assigned and methods used. Provide in the source data disaggregated sex and gender data where this information has been collected, and consent has been obtained for sharing of individual-level data; provide overall numbers in this Reporting Summary. Please state if this information has not been collected. Report sex- and gender-based analyses where performed, justify reasons for lack of sex- and gender-based analysis.* |
| Population characteristics | *Describe the covariate-relevant population characteristics of the human research participants (e.g. age, genotypic information, past and current diagnosis and treatment categories). If you filled out the behavioural & social sciences study design questions and have nothing to add here, write "See above."* |
| Recruitment | *Describe how participants were recruited. Outline any potential self-selection bias or other biases that may be present and how these are likely to impact results.* |
| Ethics oversight | *Identify the organization(s) that approved the study protocol.* |

Note that full information on the approval of the study protocol must also be provided in the manuscript.

# Field-specific reporting

Please select the one below that is the best fit for your research. If you are not sure, read the appropriate sections before making your selection.

☒ Life sciences ☐ Behavioural & social sciences ☐ Ecological, evolutionary & environmental sciences

For a reference copy of the document with all sections, see nature.com/documents/nr-reporting-summary-flat.pdf

# Life sciences study design

All studies must disclose on these points even when the disclosure is negative.

| | |
|---|---|
| Sample size | Depending on experiment (see manuscript); Sample size, number of replicates, errors bars and statistical tests were chosen based on experience and common practice in the field, and stated in each figure legend. Unless otherwise indicated, all experiments included technical replicates and were repeated at least two times. At least three different batches of Xenopus eggs were used. |
| Data exclusions | No data were excluded from analysis in this study. |
| Replication | At least two times (depending on experiments), for Xenopus studies at least three replicates were analyzed. All experimental data were consistent in all replicates. |
| Randomization | Cells were allocated based on their clear genotype without using specific randomization methods. Samples were harvested, processed and analyzed in a random order. Xenopus work: Allocation was performed randomly. |

Blinding

No blinding was applied in this study, except for migration assays where the evaluation was done blindly by a group member not involved in this project. All other data were derived from objective quantitative methods with at least two independent, successful replicates.

# Reporting for specific materials, systems and methods

We require information from authors about some types of materials, experimental systems and methods used in many studies. Here, indicate whether each material, system or method listed is relevant to your study. If you are not sure if a list item applies to your research, read the appropriate section before selecting a response.

## Materials & experimental systems

| n/a | Involved in the study |
|---|---|
| ☐ | ☒ Antibodies |
| ☐ | ☒ Eukaryotic cell lines |
| ☒ | ☐ Palaeontology and archaeology |
| ☐ | ☒ Animals and other organisms |
| ☒ | ☐ Clinical data |
| ☒ | ☐ Dual use research of concern |

## Methods

| n/a | Involved in the study |
|---|---|
| ☐ | ☒ ChIP-seq |
| ☒ | ☐ Flow cytometry |
| ☒ | ☐ MRI-based neuroimaging |

## Antibodies

Antibodies used

See also Supplemental Table 4
α-HMG20B, Rabbit, Proteintech, 14582-1-AP; α-HMG20A, Rabbit, Proteintech, 12085-1-AP; α-GSE1, Rabbit, Proteintech, 24947-1-AP; α-PWWP2A, Rabbit, Norvusbio, NBP2-13833; α-H3, Rabbit, abcam , ab1791; α-BRD2, Rabbit, Proteintech, 22236-1-AP; α-GFP, Mouse, Roche, 11814460001; α-GFP, Rabbit, abcam, ab290; α-RBBP4, Rabbit, abcam, ab488; α-MTA, Rabbit, abcam , ab71153; α-FLAG, Mouse, Sigma-Aldrich, F3165; α-FLAG (HRP), Mouse, Sigma-Aldrich , A8592; α-HA (6E2) (HRP), Mouse, Cell Signalling Technology, 2999; α-HDAC1 (10E2) (HRP), Mouse, Cell Signalling Technology, 59581; α-GFP (D5.1), Rabbit, Cell signalling Technology, 2956; α-Rabbit IgG H&L (HRP), Goat, abcam , ab97051; α-HDAC2, Mouse, abcam, ab124974; 13-0041; α-IgG , Rabbit, Epicypher, 13-0042; α-Mouse, HRP, Goat, Thermo Fisher Scientific, 31430; α-Rabbit HRP, Goat, Thermo Fisher Scientific, 31460; α-Rabbit-Alexa 488, Goat, Thermo Fisher Scientific, A-11070; α-Rabbit-Alexa 594, Goat, Thermo Fisher Scientific, A-11012

Validation

All used antibodies are commercially available. Based on the informations from manufactures' websites, all primary antibodies were validated at the companies. For the HMG20A antibody: we used hmg20a DP mESCs and PWWP2A RNAi HeLaK cells. Xenopus: primary antibody anti-Collagen Type II (DSHB, II-II6B3): Positive Tested Species Reactivity: Avian, Bovine, Broad species, Chicken, Fish, Goat, Human, Mouse, Ovine, Quail, Rabbit, Rat, Shark, Xenopus, Zebrafish; Recommended Applications: FFPE, Immunofluorescence, Immunohistochemistry, Western Blot
Validation specifics:
HMG20B antibody was validated via Western Blot of endogenous protein in human Hela cells, including RNAi, Immunoprecipitation, Immunohistochemistry and Immunofluorecence. (https://www.ptglab.com/products/pictures/pdf/14582-1-AP.pdf)
HMG20A antibody was validated via Western Blot of endogenous protein in human Hela and Jurkat cells, including RNAi, Immunoprecipitation, Immunohistochemistry and Immunofluorecence in human HepG2 cells (https://www.ptglab.com/products/pictures/pdf/12085-2-AP.pdf)
GSE1 antibody was validated via Western Blot of endogenous protein in various cell lines, Immunohistochemistry (mouse testis) and Immunofluorecence in human U2OS (https://www.ptglab.com/products/pictures/pdf/24947-1-AP.pdf)
PWWP2A antibody was validated via Immunohistochemistry and Immunofluorecence in human cell lines. PWWP2A antibody has been validated in RNAi (WB, Human)(Link et al. 2018)
H3 antibody:
Reacts with: Mouse, Rat, Human, Saccharomyces cerevisiae, Xenopus laevis, Arabidopsis thaliana, Drosophila melanogaster, Indian muntjac, Schizosaccharomyces pombe
Predicted to work with: Chicken, Dog, Caenorhabditis elegans, Ferret, Zebrafish, a wide range of other species, Mammals, Silk worm, Dictyostelium discoideum, Rainbow trout, Neurospora crassa, Toxoplasma gondii, Rice, Schistosoma mansoni, Candida albicans, Cyanidioschyzon merolaeValidated in ICC,IHC-P, ChIP, IP, WB (1/1000 - 1/5000. Detects a band of approximately 17 kDa (predicted molecular weight: 15 kDa).Can be blocked withHuman Histone H3 peptide (ab12149).) (https://www.abcam.com/histone-h3-antibody-nuclear-marker-and-chip-grade-ab1791.pdf)
Brd2 antibody was validated via Western Blot of endogenous protein in Hela cells, Immunoprecipitation, and Immunofluorecence. (https://www.ptglab.com/products/pictures/pdf/22236-1-AP.pdf) Knock down validation is reported in doi: 10.3389/fonc.2020.565820
Anti GFP roche (11814460001): Mixture of two monoclonal antibodies, supplied as a white lyophilizate containing 200µg of total Anti-GFP IgG.
Anti-GFP is tested for functionality and purity relative to a reference standard to confirm the quality of each new reagent preparation. (For more information see https://www.citeab.com/antibodies/8906830-11814460001-anti-gfp)
α-GFP (ab290):
Anti-GFP antibody (ab290) is a highly versatile antibody that gives a stronger signal than other anti-GFP antibodies available. On Western blot the antibody detects the GFP fraction from cell extracts expressing recombinant GFP fusion proteins and has also been shown to be useful on mouse sections fixed with formalin. In Immunocytochemistry, the antibody gives a very good signal on recombinant YES-GFP chimeras expressed in COS cells (McCabe et al. 1999 and figure below). It is routinely used in Immunoprecipitation (IP) and IP-Western protocols and has been used successfully in HRP Immunohistochemistry at 1:200 on whole-mount mouse embryos.

GFP antibody is reactive against all variants of Aequorea victoria GFP such as S65T-GFP, RS-GFP, YFP, CFP, RFP and EGFP.
RBPP4 antibody was validated via Western Blot of endogenous protein in human SW480 cells and female mES cells (https://www.abcam.com/rbbp4-antibody-11g10-ab488.html)
MTA1 antibody was validated via Westernblot of endogenous protein in human Help, HEK293T, Jurkat and TCMK cells, Immunohistochemistry was performed in human non-small cell lung cancer tissue Immunoprecipitation was validated in human Hela cells (https://www.abcam.com/mta1-antibody-ab71153.html)
FLAG and FLAG-HRP
Monoclonal ANTI-FLAG®M2 antibody produced in mouse has been used in: immunoblotting, immunoprecipitation, immunocytochemistry, immunofluorescence, ELISA, EIA, chromatin immunoprecipitation, electron microscopy, flow cytometry, supershift assays (https://www.sigmaaldrich.com/DE/en/product/sigma/f3165)
HA-HRP antibody was validated via Western blot analysis of extracts from COS cells (https://www.cellsignal.de/products/antibody-conjugates/ha-tag-6e2-mouse-mab-hrp-conjugate/2999)
HDAC1-AB was validated via Western blot analysis of extracts from human HeLa and mouse 3T3 cells (https://www.cellsignal.de/products/antibody-conjugates/hdac1-10e2-mouse-mab-hrp-conjugate/59581)
GFP (D5.1) was validated via Western blot and Immunohistochemistry analysis of extracts from HCC827 cells transfected with GFP (https://www.cellsignal.de/products/primary-antibodies/gfp-d5-1-rabbit-mab/2956)
HDAC2 (ab124974) antibody was validated via western blot in various human (HDAC2 Knockout) cell lines. ChIP was validated in human Hela cells (https://www.abcam.com/hdac2-antibody-epr5001-chip-grade-ab124974.html)
H3K4me3 antibody was validated via SNAP specificity analysis in CUT&RUN in K562 cells, Immunofluorescence In hela cells, Western blot validations performed in whole cell extracts from HeLa, Hep G2, HCT 116, MCF7, U-2 OS, A549, HEK-293, NIH/3T3, and PC-12 cells (https://www.epicypher.com/content/documents/tds/13-0041.pdf)
Lack of α-IgG antibody specificity was validated via CUT&RUN genome wide enrichment. Functional binding to Protein A was validated in Protein A immunoprecipitation (https://www.epicypher.com/content/documents/tds/13-0042.pdf)

## Eukaryotic cell lines

Policy information about cell lines and Sex and Gender in Research

| | |
|---|---|
| Cell line source(s) | HeLa Kyoto (HeLaK) cells were a gift from Prof. Dr Heinrich Leonhardt (LMU Munich, Germany).<br>WT mESCs(v6.5) were a gift from Prof. Dr. Thomas Braun (Max Planck Institute for Heart and Lung Research, Bad Nauheim, Germany).<br>Hmg20a DP mESCs were generated in-house via CRISPR-Cas9 nickase system. |
| Authentication | No authentication was performed. |
| Mycoplasma contamination | All cells were tested regularly for Mycoplasma contaminations with PCR tests. No contamination was detected. |
| Commonly misidentified lines<br>(See ICLAC register) | No cell lines from the ICLAC register were used. |

## Animals and other research organisms

Policy information about studies involving animals; ARRIVE guidelines recommended for reporting animal research, and Sex and Gender in Research

| | |
|---|---|
| Laboratory animals | Xenopus laevis frogs were used to provide eggs (females) and testis (males). |
| Wild animals | No wild animals were used. |
| Reporting on sex | Embryos were analyzed before stages of sex determination. |
| Field-collected samples | No field-collected samples were used. |
| Ethics oversight | Embryos were obtained by in vitro fertilization using adult frogs from our colony at the University of Marbug, Germany. All procedures were performed according to the German Animal Use and Care Act (Tierschutzgesetz) and approved by the German state administration Hesse (Regierungspräsidium Giessen, approval number A 16/2017). |

Note that full information on the approval of the study protocol must also be provided in the manuscript.

## ChIP-seq

### Data deposition

☒ Confirm that both raw and final processed data have been deposited in a public database such as GEO.

☒ Confirm that you have deposited or provided access to graph files (e.g. BED files) for the called peaks.

| | |
|---|---|
| Data access links<br>*May remain private before publication.* | GEO accession: "GSE202199[https://www.ncbi.nlm.nih.gov/geo/query/acc.cgi?acc=GSE202199]" |
| Files in database submission | Cut&Run (mouse):<br>GSM6102982 HMG20AKO Day2_HMG20A CNR_Rep1<br>GSM6102982_KON1HMG_12_1_val_1.fq.gz.hisat.bam_rm_dupli.bam_bigwig.bw |

GSM6102983 HMG20AKO Day2_IgG CNR_Rep1
GSM6102983_KON1IGG_10_1_val_1.fq.gz.hisat.bam_rm_dupli.bam_bigwig.bw
GSM6102984 HMG20AKO Day2_HMG20A CNR_Rep2
GSM6102984_KON2HMG_16_1_val_1.fq.gz.hisat.bam_rm_dupli.bam_bigwig.bw
GSM6102985 HMG20AKO Day2_IgG CNR_Rep2
GSM6102985_KON2IGG_14_1_val_1.fq.gz.hisat.bam_rm_dupli.bam_bigwig.bw
GSM6102986 WT Day2_HMG20A CNR_Rep1
GSM6102986_WTN1HMG_4_1_val_1.fq.gz.hisat.bam_rm_dupli.bam_bigwig.bw
GSM6102987 WT Day2_IgG CNR_Rep1 GSM6102987_WTN1IGG_2_1_val_1.fq.gz.hisat.bam_rm_dupli.bam_bigwig.bw
GSM6102988 WT Day2_HMG20A CNR_Rep2
GSM6102988_WTN2HMG_8_1_val_1.fq.gz.hisat.bam_rm_dupli.bam_bigwig.bw
GSM6102989 WT Day2_IgG CNR_Rep2 GSM6102989_WTN2IGG_6_1_val_1.fq.gz.hisat.bam_rm_dupli.bam_bigwig.bw

ATAC-seq (mouse)
GSM6710659 Primed Hmg20a DP #26 rep1 GSM6710659_KO_PR_1_1_bigwig.bw
GSM6710660 Primed Hmg20a DP #26 rep2 GSM6710660_KO_PR_2_1_bigwig.bw
GSM6710661 Primed wild type rep1 GSM6710661_WT_PR_1_1_bigwig.bw
GSM6710662 Primed wild type rep2 GSM6710662_WT_PR_2_1_bigwig.bw

ChIP-seq (human)
GSM6102990 HeLa_GFP-HMG20A_input GSM6102990_GIGENAH003.fastq_hg19_sorted.bam_min.bam.bigwig
GSM6102991 HeLa_GFP_ChIP GSM6102991_GIGENAH006.fastq_hg19_sorted.bam_min.bam.bigwig
GSM6102992 HeLa_GFP-HMG20A_ChIP_Rep1 GSM6102992_GIGENAH008.fastq_hg19_sorted.bam_min.bam.bigwig
GSM6102993 HeLa_GFP-HMG20A_ChIP_Rep2 GSM6102993_GIGENAH009.fastq_hg19_sorted.bam_min.bam.bigwig

Genome browser session
(e.g. UCSC)

https://genome.ucsc.edu/s/MarekB/hg19_AH_NC

https://genome-euro.ucsc.edu/s/MarekB/mm9_AH_CnR_ATAC

## Methodology

Replicates

Two

Sequencing depth

ChIP-seq (human)
GSM6102990 HeLa_GFP-HMG20A_input 39796591 single-end reads
GSM6102991 HeLa_GFP_ChIP 37245806 single-end reads
GSM6102992 HeLa_GFP-HMG20A_ChIP_Rep1 26629040 single-end reads
GSM6102993 HeLa_GFP-HMG20A_ChIP_Rep2 51635583 single-end reads

ATAC-seq (mouse)
GSM6710659 Primed Hmg20a DP #26 rep1 35238499 paired-end reads
GSM6710660 Primed Hmg20a DP #26 rep2 36509720 paired-end reads
GSM6710661 Primed wild type rep1 31753067 paired-end reads
GSM6710662 Primed wild type rep2 30977579 paired-end reads

Cut&Run (mouse):
GSM6102982 HMG20AKO Day2_HMG20A CNR_Rep1 11761245 paired-end reads
GSM6102983 HMG20AKO Day2_IgG CNR_Rep1 22264863 paired-end reads
GSM6102984 HMG20AKO Day2_HMG20A CNR_Rep2 13085659 paired-end reads
GSM6102985 HMG20AKO Day2_IgG CNR_Rep2 14723116 paired-end reads
GSM6102986 WT Day2_HMG20A CNR_Rep1 12945500 paired-end reads
GSM6102987 WT Day2_IgG CNR_Rep1 14834091 paired-end reads
GSM6102988 WT Day2_HMG20A CNR_Rep2 11661060 paired-end reads
GSM6102989 WT Day2_IgG CNR_Rep2 18566447 paired-end reads

Antibodies

α-GFP, Rabbit, abcam, ab290 (ChIP-seq); α-HMG20A, Rabbit, Proteintech, 12085-1-AP (CUT&RUN); α-IgG , Rabbit, Epicypher, 13-0042 (CUT&RUN)

Peak calling parameters

Peak calling was done using MACS2 using default parameters (-q 0.05 -g 2.7e9 (human) 2.2e9 (mouse)). The resulting set was filtered against blacklisted chromatin regions, as detected by ENCODE. Consensus peak sets were calculated by GenomicRanges' reduce function. Read counts across peaks were determined using the featureCounts function of the Subread package. Differential binding analysis was performed by using DESeq2 after merging overlapping peaks into reference peak sets, using the reduce function of the BioConductor GenomicRanges package.

Data quality

Raw FASTQ files were inspected with fastqc. Trim_galore was used for quality aware trimming and removal of adapters using default parameters. Mapping and peak calling were evaluated by optical inspection in the genome browser as well as pair-wise correlation analysis of binding profiles (coverage).

Software

FastQC (https://www.bioinformatics.babraham.ac.uk/projects/fastqc/)
Trim Galore (https://www.bioinformatics.babraham.ac.uk/projects/trim_galore/)
BWT index from Illumina's iGenome repository (https://emea.support.illumina.com/sequencing/, sequencing_software/igenome.html)
bowtie version 1.1.2

```
hisat2 v.2.2.171

Integrative Genome Viewer (IGV),
MACS2 2.2.7.1
R version 4.1.2 (2021-11-01)
Platform: x86_64-apple-darwin17.0 (64-bit)
Running under: macOS Catalina 10.15.7

Matrix products: default
BLAS:   /System/Library/Frameworks/Accelerate.framework/Versions/A/Frameworks/vecLib.framework/Versions/A/libBLAS.dylib
LAPACK: /Library/Frameworks/R.framework/Versions/4.1/Resources/lib/libRlapack.dylib

Random number generation:
 RNG:     Mersenne-Twister
 Normal:  Inversion
 Sample:  Rounding

locale:
[1] en_US.UTF-8/en_US.UTF-8/en_US.UTF-8/C/en_US.UTF-8/en_US.UTF-8

attached base packages:
[1] grid      stats4    stats     graphics  grDevices utils     datasets  methods   base

other attached packages:
 [1] clusterProfiler_4.2.2       GeneOverlap_1.30.0          data.table_1.14.2           Hmisc_4.6-0
 [5] ggplot2_3.3.5               Formula_1.2-4               survival_3.2-13             lattice_0.20-45
 [9] fields_13.3                 viridis_0.6.2               viridisLite_0.4.0           spam_2.8-0
[13] DESeq2_1.34.0               limma_3.50.0                fts_0.9.9.2                 zoo_1.8-9
[17] rtracklayer_1.54.0          gplots_3.1.1                RColorBrewer_1.1-2          Gviz_1.38.3
[21] ShortRead_1.52.0            GenomicAlignments_1.30.0    SummarizedExperiment_1.24.0 MatrixGenerics_1.6.0
[25] matrixStats_0.61.0          Rsamtools_2.10.0            Biostrings_2.62.0           XVector_0.34.0
[29] BiocParallel_1.28.3         GenomicFeatures_1.46.4      AnnotationDbi_1.56.2        Biobase_2.54.0
[33] GenomicRanges_1.46.1        GenomeInfoDb_1.30.1         IRanges_2.28.0              S4Vectors_0.32.3
[37] BiocGenerics_0.40.0

loaded via a namespace (and not attached):
  [1] utf8_1.2.2             tidyselect_1.1.1       RSQLite_2.2.9          htmlwidgets_1.5.4      scatterpie_0.1.7
  [6] munsell_0.5.0          withr_2.4.3            colorspace_2.0-2       GOSemSim_2.20.0        filelock_1.0.2
 [11] knitr_1.37             rstudioapi_0.13        DOSE_3.20.1            GenomeInfoDbData_1.2.7 hwriter_1.3.2
 [16] polyclip_1.10-0        bit64_4.0.5            farver_2.1.0           downloader_0.4         vctrs_0.3.8
 [21] treeio_1.18.1          generics_0.1.2         xfun_0.29              biovizBase_1.42.0      BiocFileCache_2.2.1
 [26] R6_2.5.1               graphlayouts_0.8.0     locfit_1.5-9.4         AnnotationFilter_1.18.0 bitops_1.0-7
 [31] cachem_1.0.6           fgsea_1.20.0           gridGraphics_0.5-1     DelayedArray_0.20.0    assertthat_0.2.1
 [36] BiocIO_1.4.0           scales_1.1.1           ggraph_2.0.5           nnet_7.3-17            enrichplot_1.14.1
 [41] gtable_0.3.0           tidygraph_1.2.0        ensembldb_2.18.3       rlang_1.0.1            genefilter_1.76.0
 [46] splines_4.1.2          lazyeval_0.2.2         GEOquery_2.62.2        dichromat_2.0-0        checkmate_2.0.0
 [51] yaml_2.2.2             reshape2_1.4.4         backports_1.4.1        qvalue_2.26.0          tools_4.1.2
 [56] ggplotify_0.1.0        ellipsis_0.3.2         Rcpp_1.0.8             plyr_1.8.6             base64enc_0.1-3
 [61] progress_1.2.2         zlibbioc_1.40.0        purrr_0.3.4            RCurl_1.98-1.5         prettyunits_1.1.1
 [66] rpart_4.1.16           ggrepel_0.9.1          cluster_2.1.2          magrittr_2.0.2         DO.db_2.9
 [71] ProtGenerics_1.26.0    hms_1.1.1              patchwork_1.1.1        xtable_1.8-4           XML_3.99-0.8
 [76] jpeg_0.1-9             gridExtra_2.3          compiler_4.1.2         biomaRt_2.50.3         tibble_3.1.6
 [81] maps_3.4.0             shadowtext_0.1.1       KernSmooth_2.23-20     crayon_1.4.2           htmltools_0.5.2
 [86] ggfun_0.0.5            tzdb_0.2.0             tidyr_1.2.0            geneplotter_1.72.0     aplot_0.1.2
 [91] DBI_1.1.2              tweenr_1.0.2           dbplyr_2.1.1           MASS_7.3-55            rappdirs_0.3.3
 [96] Matrix_1.4-0           readr_2.1.2            cli_3.1.1              parallel_4.1.2         dotCall64_1.0-1
[101] igraph_1.2.11          pkgconfig_2.0.3        foreign_0.8-82         xml2_1.3.3             ggtree_3.2.1
[106] annotate_1.72.0        yulab.utils_0.0.4      stringr_1.4.0          VariantAnnotation_1.40.0 digest_0.6.29
[111] fastmatch_1.1-3        tidytree_0.3.7         htmlTable_2.4.0        restfulr_0.0.13        curl_4.3.2
[116] gtools_3.9.2           rjson_0.2.21           jsonlite_1.7.3         lifecycle_1.0.1        nlme_3.1-155
[121] BSgenome_1.62.0        fansi_1.0.2            pillar_1.7.0           KEGGREST_1.34.0        fastmap_1.1.0
[126] httr_1.4.2             GO.db_3.14.0           glue_1.6.1             png_0.1-7              bit_4.0.4
[131] ggforce_0.3.3          stringi_1.7.6          blob_1.2.2             latticeExtra_0.6-29    caTools_1.18.2
[136] memoise_2.0.1          dplyr_1.0.7            ape_5.6-1
```
